# Supplementary material for: Correlated optical convolutional neural network with “quantum speedup”
Source: Light Sci Appl. 2024 Jan 31;13:36. doi: 10.1038/s41377-024-01376-7 (PMC10828439; doi:10.1038/s41377-024-01376-7)
Supplement: Supplementary file 1 — SP [file 41377_2024_1376_MOESM1_ESM.docx]

**Supplementary Information for Correlated Optical Convolutional Neural Network with “Quantum Speedup”**

Yifan Sun, Qian Li, Ling-Jun Kong, and Xiangdong Zhang*

*Key Laboratory of advanced optoelectronic quantum architecture and measurements of Ministry of Education, Beijing Key Laboratory of Nanophotonics & Ultrafine Optoelectronic Systems, School of Physics, Beijing Institute of Technology, 100081 Beijing, China.*

**Author to whom any correspondence should be addressed: zhangxd@bit.edu.cn*

**S1. The optical modulation unit (OMU) for the 2-beam operations in the part of the convolution**

According to the main text, the 2-beam operation can in principle generate any kind of correlated state of a pair of beams. Because the correlations of the beams form a Hilbert space, the operations that address all the correlations are actually the elements of a unitary group. Using the decomposition of arbitrary unitary operators [1,2], the 2-beam operations we consider can be realized by two kinds of basic arrangements. One is the unitary rotation of the polarization of a single beam. As we mentioned in the main text, it can be realized by a setup two quarter-wave plates (QWPs) and one half-wave plate (HWP), also known as the Q-H-Q and widely discussed. The other arrangement is an analogy of the quantum CZ operation, which is called an optical modulation unit (OMU) in the main text. We next go into the details of the OMU. Consider two correlated beams, say $\boldsymbol{E}_{1}$ and $\boldsymbol{E}_{2}$, and their state is denoted by $\left| 2E \right)\boldsymbol{=}c_{h_{1}h_{2}}\left| \boldsymbol{h}_{1} \right)\left| \boldsymbol{h}_{2} \right)\boldsymbol{+}c_{h_{1}v_{2}}\left| \boldsymbol{h}_{1} \right)\left| \boldsymbol{v}_{2} \right)\boldsymbol{+}c_{v_{1}h_{2}}\left| \boldsymbol{v}_{1} \right)\left| \boldsymbol{h}_{2} \right)\boldsymbol{+}c_{v_{1}v_{2}}\left| \boldsymbol{v}_{1} \right)\left| \boldsymbol{v}_{2} \right)$. The function of the OMU is flipping the phase of $c_{v_{1}v_{2}}\left| \boldsymbol{v}_{1} \right)\left| \boldsymbol{v}_{2} \right)$, such that $c_{v_{1}v_{2}}\left| \boldsymbol{v}_{1} \right)\left| \boldsymbol{v}_{2} \right)\boldsymbol{\to-}c_{v_{1}v_{2}}\left| \boldsymbol{v}_{1} \right)\left| \boldsymbol{v}_{2} \right)$. Without loss of generality, consider Eq. (5) in the main text when $M=2$, one has

$$\boldsymbol{E}_{1}=f_{1}\left( p_{1,1}^{H}\boldsymbol{h}+p_{1,1}^{V}\boldsymbol{v} \right)+f_{2}\left( p_{1,2}^{H}\boldsymbol{h}+p_{1,2}^{V}\boldsymbol{v} \right)$$

$$\boldsymbol{E}_{2}=f_{1}\left( p_{2,1}^{H}\boldsymbol{h}+p_{2,1}^{V}\boldsymbol{v} \right)+f_{2}\left( p_{2,2}^{H}\boldsymbol{h}+p_{2,2}^{V}\boldsymbol{v} \right) (S1)$$

Then, the correlations can be given by

$$c_{h_{1}h_{2}}=\int\left( {\boldsymbol{h}\boldsymbol{\cdot}\boldsymbol{E}}_{1} \right)\left( {\boldsymbol{h}\boldsymbol{\cdot}\boldsymbol{E}}_{2} \right)d\Omega=p_{1,1}^{H}p_{2,1}^{H}+p_{1,1}^{H}p_{2,1}^{H}$$

$$c_{h_{1}v_{2}}=\int\left( {\boldsymbol{v}\boldsymbol{\cdot}\boldsymbol{E}}_{1} \right)\left( {\boldsymbol{h}\boldsymbol{\cdot}\boldsymbol{E}}_{2} \right)d\Omega=p_{1,1}^{V}p_{2,1}^{H}+p_{1,1}^{V}p_{2,1}^{H}$$

$$c_{v_{1}h_{2}}=\int\left( {\boldsymbol{h}\boldsymbol{\cdot}\boldsymbol{E}}_{1} \right)\left( {\boldsymbol{v}\boldsymbol{\cdot}\boldsymbol{E}}_{2} \right)d\Omega=p_{1,1}^{V}p_{2,1}^{H}+p_{1,1}^{V}p_{2,1}^{H}$$

$$c_{v_{1}v_{2}}=\int\left( {\boldsymbol{v}\boldsymbol{\cdot}\boldsymbol{E}}_{1} \right)\left( {\boldsymbol{v}\boldsymbol{\cdot}\boldsymbol{E}}_{2} \right)d\Omega=p_{1,1}^{V}p_{2,1}^{V}+p_{1,1}^{V}p_{2,1}^{V} (S2)$$

For implementing the OMU, consider the operation ${OC}_{n}$ on the $m$th beam,

$${OC}_{n}\boldsymbol{h}\boldsymbol{=}\exp\left( i\omega_{n,H}t \right)\boldsymbol{h}\boldsymbol{,}{OC}_{n}\boldsymbol{v}\boldsymbol{=}\exp\left( i\omega_{n,V}t \right)\boldsymbol{v} \left( S3 \right)$$

Eq. (S3) means that ${OC}_{n}$ loads an oscillation with a frequency $\omega_{n,H}$ ($\omega_{n,V}$) on the horizontal (vertical) polarization component. This can be implemented by a polarization-dependent shutter, modulating the beam at a rather low frequency. Thus, the oscillation indicated by Eq. (S3) can be observed by an oscilloscope, and further processed. Apply the operation on the two beams, one has

$${OC}_{1}\boldsymbol{E}_{1}=f_{1}\left( e^{i\omega_{1,H}t}p_{1,1}^{H}\boldsymbol{h}+e^{i\omega_{1,V}t}p_{1,1}^{V}\boldsymbol{v} \right)+f_{2}\left( e^{i\omega_{1,H}t}p_{1,2}^{H}\boldsymbol{h}+e^{i\omega_{1,V}t}p_{1,2}^{V}\boldsymbol{v} \right)$$

$${OC}_{2}\boldsymbol{E}_{2}=f_{1}\left( e^{i\omega_{2,H}t}p_{2,1}^{H}\boldsymbol{h}+e^{i\omega_{2,V}t}p_{2,1}^{V}\boldsymbol{v} \right)+f_{2}\left( e^{i\omega_{2,H}t}p_{2,2}^{H}\boldsymbol{h}+e^{i\omega_{2,V}t}p_{2,2}^{V}\boldsymbol{v} \right) \left( S4 \right)$$

Then,

$$\int\left( \boldsymbol{h}\boldsymbol{\cdot}{OC}_{1}\boldsymbol{E}_{1} \right)\left( \boldsymbol{h}\boldsymbol{\cdot}{OC}_{2}\boldsymbol{E}_{2} \right)d\Omega=e^{i\left( \omega_{1,H}+\omega_{2,H} \right)t}\left( p_{1,1}^{H}p_{2,1}^{H}+p_{1,1}^{H}p_{2,1}^{H} \right)$$

$$\int\left( \boldsymbol{v}\boldsymbol{\cdot}{OC}_{1}\boldsymbol{E}_{1} \right)\left( \boldsymbol{h}\boldsymbol{\cdot}{OC}_{2}\boldsymbol{E}_{2} \right)d\Omega=e^{i\left( \omega_{1,H}+\omega_{2,V} \right)t}\left( p_{1,1}^{V}p_{2,1}^{H}+p_{1,1}^{V}p_{2,1}^{H} \right)$$

$$\int\left( \boldsymbol{h}\boldsymbol{\cdot}{OC}_{1}\boldsymbol{E}_{1} \right)\left( \boldsymbol{v}\boldsymbol{\cdot}{OC}_{2}\boldsymbol{E}_{2} \right)d\Omega=e^{i\left( \omega_{1,V}+\omega_{2,H} \right)t}\left( p_{1,1}^{H}p_{2,1}^{V}+p_{1,1}^{H}p_{2,1}^{V} \right)$$

$$\int\left( \boldsymbol{v}\boldsymbol{\cdot}{OC}_{1}\boldsymbol{E}_{1} \right)\left( \boldsymbol{v}\boldsymbol{\cdot}{OC}_{2}\boldsymbol{E}_{2} \right)d\Omega=e^{i\left( \omega_{1,V}+\omega_{2,V} \right)t}\left( p_{1,1}^{V}p_{2,1}^{V}+p_{1,1}^{V}p_{2,1}^{V} \right) \left( S5 \right)$$

Without loss of generality, one can set the value of the frequencies (e.g., let $\omega_{1,V}>\omega_{1,H}$ and $\omega_{2,V}>\omega_{2,H}$) such that the above correlations can be distinguished. Hence, a frequency-dependent phase shift in the above correlation, shifting the $\omega_{1,V}+\omega_{2,V}$ component by $\pi$. Then, taking the complex amplitude of the oscillation, one can effectively has the transformation

$$c_{h_{1}h_{2}}\to c_{h_{1}h_{2}}, c_{h_{1}v_{2}}\to c_{h_{1}v_{2}}, c_{v_{1}h_{2}}\to c_{v_{1}h_{2}}, c_{v_{1}v_{2}}\to-c_{v_{1}v_{2}} \left( S6 \right)$$

equivalent to the CZ gate in quantum computing. Notice that the phase shifting operation is permitted to be performed in the part of processing the signals, because the factor $e^{i\left( \omega_{1,V}+\omega_{2,V} \right)t}$ will not be affected by other operations in the network. A natural consequence of the proposal is that the number of the implementable OMUs is limited by the capability of setting distinguishable frequencies, such as that required by distinguishing $\omega_{1,V}+\omega_{2,V}$. In other words, it is limited by the bandwidth of the oscillation tuning by operation ${OC}_{m}$. In a more general case, the proposal can also be satisfied. Consider the $N$-qubit state given by Eq. (9) and the conditions given by Eq. (10) in the main text, and denote the $N$ beams for mimicking Eq. (9) by $\boldsymbol{E}_{n}$ ($n\boldsymbol{=}1\boldsymbol{,\ldots,}N$). The OMU on the $i$th beam $\boldsymbol{E}_{i}$ and $j$th beam $\boldsymbol{E}_{j}$ can be given by the above operation,

$${OC}_{i}\boldsymbol{E}_{i}=\sum_{k=1}^{M} f_{k}\left( e^{i\omega_{i,H}t}p_{i,k}^{H}\boldsymbol{h}+e^{i\omega_{i,V}t}p_{i,k}^{V}\boldsymbol{v} \right), {OC}_{j}\boldsymbol{E}_{j}=\sum_{k=1}^{M} f_{k}\left( e^{i\omega_{j,H}t}p_{j,k}^{H}\boldsymbol{h}+e^{i\omega_{j,V}t}p_{j,k}^{V}\boldsymbol{v} \right) \left( S7 \right)$$

Then, the related correlations are,

$$e^{i\left( \omega_{i,H}+\omega_{j,H} \right)t}\sum_{k=1}^{M} p_{1,k}^{O_{1}}\boldsymbol{\ldots}p_{i,k}^{H}\boldsymbol{\ldots}p_{j,k}^{H}\boldsymbol{\ldots}p_{N,k}^{O_{N}}=e^{i\left( \omega_{i,H}+\omega_{j,H} \right)t}c_{o_{1}\ldots h_{i}\ldots h_{j}\ldots o_{N}}$$

$$e^{i\left( \omega_{i,H}+\omega_{j,V} \right)t}\sum_{k=1}^{M} p_{1,k}^{O_{1}}\boldsymbol{\ldots}p_{i,k}^{H}\boldsymbol{\ldots}p_{j,k}^{V}\boldsymbol{\ldots}p_{N,k}^{O_{N}}=e^{i\left( \omega_{i,H}+\omega_{j,V} \right)t}c_{o_{1}\ldots h_{i}\ldots v_{j}\ldots o_{N}}$$

$$e^{i\left( \omega_{i,V}+\omega_{j,H} \right)t}\sum_{k=1}^{M} p_{1,k}^{O_{1}}\boldsymbol{\ldots}p_{i,k}^{V}\boldsymbol{\ldots}p_{j,k}^{H}\boldsymbol{\ldots}p_{N,k}^{O_{N}}=e^{i\left( \omega_{i,V}+\omega_{j,H} \right)t}c_{o_{1}\ldots v_{i}\ldots h_{j}\ldots o_{N}}$$

$$e^{i\left( \omega_{i,V}+\omega_{j,V} \right)t}\sum_{k=1}^{M} p_{1,k}^{O_{1}}\boldsymbol{\ldots}p_{i,k}^{V}\boldsymbol{\ldots}p_{j,k}^{V}\boldsymbol{\ldots}p_{N,k}^{O_{N}}=e^{i\left( \omega_{i,V}+\omega_{j,V} \right)t}c_{o_{1}\ldots v_{i}\ldots v_{j}\ldots o_{N}} \left( S8 \right)$$

where $O_{n}$ takes $"H"$ ($"V"$) with $o_{n}=h_{n}$ ($o_{n}=v_{n}$), and $n=1,\ldots,N.$ One can also shift the $\omega_{i,V}+\omega_{j,V}$ component by $\pi$, and then obtain effective transformation,

$$c_{o_{1}\ldots h_{i}\ldots h_{j}\ldots o_{N}}\to c_{o_{1}\ldots h_{i}\ldots h_{j}\ldots o_{N}}, c_{o_{1}\ldots h_{i}\ldots v_{j}\ldots o_{N}}\to c_{o_{1}\ldots h_{i}\ldots v_{j}\ldots o_{N}}$$

$$c_{o_{1}\ldots v_{i}\ldots h_{j}\ldots\alpha_{N}}\to c_{o_{1}\ldots v_{i}\ldots h_{j}\ldots o_{N}}, c_{o_{1}\ldots v_{i}\ldots v_{j}\ldots o_{N}}\to-c_{o_{1}\ldots v_{i}\ldots v_{j}\ldots o_{N}} \left( S9 \right)$$

giving the operation analog to the CZ gate in $N$-qubit system. As mentioned in the main text and in Ref. [2], an arbitrary kind of the 2-beam correlation can be generated by 8 Q-H-Qs and 3 OMUs analog to CZ gates. Therefore, the basic block for a C-layer can be given.

**S2.** **The optical modulation unit (OMU) for the** **2-beam operations in the part of the convolution**

The key feature of the COCNN is the sharp reduction in the data size during the information processing. Such a reduction is implemented by the P-layers, which subtract the sub-space of a large Hilbert space of the beam states. We have shown that the setup leads to a steeper gradient of the loss function. In this section, we show the details of basic block, or the combiner in the main text, for a P-layer. As a simple example, we firstly consider the three-beam case when $M=4$, expressed by

$$\boldsymbol{E}_{1}=\left( p_{1,1}^{H}\boldsymbol{h}+p_{1,1}^{V}\boldsymbol{v} \right)f_{1}+\left( p_{1,2}^{H}\boldsymbol{h}+p_{1,2}^{V}\boldsymbol{v} \right)f_{2}+\left( p_{1,3}^{H}\boldsymbol{h}+p_{1,3}^{V}\boldsymbol{v} \right)f_{3}+\left( p_{1,4}^{H}\boldsymbol{h}+p_{1,4}^{V}\boldsymbol{v} \right)f_{4}$$

$$\boldsymbol{E}_{2}=\left( p_{2,1}^{H}\boldsymbol{h}+p_{2,1}^{V}\boldsymbol{v} \right)f_{1}+\left( p_{2,2}^{H}\boldsymbol{h}+p_{2,2}^{V}\boldsymbol{v} \right)f_{2}+\left( p_{2,3}^{H}\boldsymbol{h}+p_{2,3}^{V}\boldsymbol{v} \right)f_{3}+\left( p_{2,4}^{H}\boldsymbol{h}+p_{2,4}^{V}\boldsymbol{v} \right)f_{4}$$

$$\boldsymbol{E}_{3}=\left( p_{3,1}^{H}\boldsymbol{h}+p_{3,1}^{V}\boldsymbol{v} \right)f_{1}+\left( p_{3,2}^{H}\boldsymbol{h}+p_{3,2}^{V}\boldsymbol{v} \right)f_{2}+\left( p_{3,3}^{H}\boldsymbol{h}+p_{3,3}^{V}\boldsymbol{v} \right)f_{3}+\left( p_{3,4}^{H}\boldsymbol{h}+p_{3,4}^{V}\boldsymbol{v} \right)f_{4}$$

$$\left( S10 \right)$$

In order to pool the data encoded by the beams, or equivalently in this example, to encode the correlation of the three beams in only $\boldsymbol{E}_{2}$ and $\boldsymbol{E}_{3}$, one firstly performs the following action on $\boldsymbol{E}_{1}$,

$$\boldsymbol{E}_{1}\to\boldsymbol{E}_{1}^{'}=\left( \delta_{H}p_{1,1}^{H}\boldsymbol{+}\delta_{V}p_{1,1}^{V} \right)\frac{\boldsymbol{h}\boldsymbol{+}\boldsymbol{v}}{\sqrt{2}}f_{1}+\left( {\delta_{H}p}_{1,2}^{H}+\delta_{V}p_{1,2}^{V} \right)\frac{\boldsymbol{h}\boldsymbol{+}\boldsymbol{v}}{\sqrt{2}}f_{2}+\left( \delta_{H}p_{1,3}^{H}+\delta_{V}p_{1,3}^{V} \right)\frac{\boldsymbol{h}\boldsymbol{+}\boldsymbol{v}}{\sqrt{2}}f_{3}+\left( \delta_{H}p_{1,4}^{H}+\delta_{V}p_{1,4}^{V} \right)\frac{\boldsymbol{h}\boldsymbol{+}\boldsymbol{v}}{\sqrt{2}}f_{4}$$

$$\left( S11 \right)$$

where $\delta_{H}$ and $\delta_{V}$ satisfy that $\delta_{H}\delta_{V}=0, \delta_{H}\delta_{H}=\delta_{V}\delta_{V}=1$. This can be implemented by introducing a random phase between the horizontal and vertical components and project them to the $45^{\circ}$ direction. In other words, the horizontal and vertical components are changed to incoherent components. Secondly, introducing nonlinear operation such that

$$\left( \boldsymbol{E}_{1}^{'},\boldsymbol{E}_{2} \right)\underset{\to}{NL}\boldsymbol{E}_{2}^{'}=\left( p_{2,1}^{H}\boldsymbol{h}+p_{2,1}^{V}\boldsymbol{v} \right)\left( \delta_{H}p_{1,1}^{H}\boldsymbol{+}\delta_{V}p_{1,1}^{V} \right)f_{1}+\left( p_{2,2}^{H}\boldsymbol{h}+p_{2,2}^{V}\boldsymbol{v} \right)\left( {\delta_{H}p}_{1,2}^{H}+\delta_{V}p_{1,2}^{V} \right)f_{2}+\left( p_{2,3}^{H}\boldsymbol{h}+p_{2,3}^{V}\boldsymbol{v} \right)\left( \delta_{H}p_{1,3}^{H}+\delta_{V}p_{1,3}^{V} \right)f_{3}+\left( p_{2,4}^{H}\boldsymbol{h}+p_{2,4}^{V}\boldsymbol{v} \right)\left( \delta_{H}p_{1,4}^{H}+\delta_{V}p_{1,4}^{V} \right)f_{4}$$

$$\left( S12 \right)$$

Such a nonlinear operation multiplies the amplitudes of corresponding modes and retain the incoherent relation. The whole setup for performing the Eq. (S11) and E. (S12) is called a combiner in the main text. Actually, the correlated state of $\boldsymbol{E}_{2}^{'}$ and $\boldsymbol{E}_{3}$ has a good correspondence with the 2-qubit state of the subsystem of three qubits, usually expressed by the partial trace $\mathrm{Tr}_{1}\left\{ \left| \psi_{3} \right\rangle\left\langle\psi_{3} \right| \right\}$. Here, $\left| \psi_{3} \right\rangle$ is an arbitrary 3-qubit state, as expressed by Eq. (15) in the main text, and $\mathrm{Tr}_{1}\left\{ \right\}$ means tracing out the first qubit. The correspondence can be seen by the measurements of the states. Define the projections of $\boldsymbol{E}_{2}^{'}$ and $\boldsymbol{E}_{3}$ by $\boldsymbol{e}_{2}=\alpha_{2}\boldsymbol{h}+\beta_{2}\boldsymbol{v}$ and $\boldsymbol{e}_{3}=\alpha_{3}\boldsymbol{h}+\beta_{3}\boldsymbol{v}$ respectively, and the corresponding measurement basis for the 2-qubit subspace by $\left| \boldsymbol{e}_{2} \right\rangle\left| \boldsymbol{e}_{3} \right\rangle=\left( \alpha_{2}\left| \boldsymbol{h} \right\rangle+\beta_{2}\left| \boldsymbol{v} \right\rangle\right)\otimes\left( \alpha_{3}\left| \boldsymbol{h} \right\rangle+\beta_{3}\left| \boldsymbol{v} \right\rangle\right)$. Using Eq. (S12) and Eq. (S10), one has the correlation

$$I\boldsymbol{=}\int\left( {\boldsymbol{e}_{2}\boldsymbol{\cdot E}}_{2}^{\boldsymbol{'}} \right)\left( {\boldsymbol{e}_{3}\boldsymbol{\cdot E}}_{3} \right)d\Omega$$

$$\boldsymbol{=}\int\left[ \boldsymbol{e}_{2}\boldsymbol{\cdot}\sum_{m=1}^{4} f_{m}\left( \delta_{H}p_{1,m}^{H}\boldsymbol{+}\delta_{V}p_{1,m}^{V} \right)\left( p_{2,m}^{H}\boldsymbol{h}+p_{2,m}^{V}\boldsymbol{v} \right) \right]\left[ \boldsymbol{e}_{3}\boldsymbol{\cdot}\sum_{m=1}^{4} f_{m}\left( p_{3,m}^{H}\boldsymbol{h}+p_{3,m}^{V}\boldsymbol{v} \right) \right]d\Omega=\sum_{\boldsymbol{O}_{2}\boldsymbol{,}\boldsymbol{O}_{3}\boldsymbol{=h}}^{\boldsymbol{v}} \sum_{m=1}^{4} \left( \delta_{H}p_{1,m}^{H}\boldsymbol{+}\delta_{V}p_{1,m}^{V} \right)p_{2,m}^{O_{2}}p_{3,m}^{O_{3}}\left( \boldsymbol{e}_{2}\boldsymbol{\cdot}\boldsymbol{O}_{2} \right)\left( \boldsymbol{e}_{3}\boldsymbol{\cdot}\boldsymbol{O}_{3} \right)$$

$$\left( S13 \right)$$

$\boldsymbol{O}_{2}$ and $\boldsymbol{O}_{3}$ can only be $\boldsymbol{h}$ or $\boldsymbol{v}$. $O_{2}$ and $O_{3}$ actually denotes the corresponding superscripts ($O_{2}$ or $O_{3}$ takes $"H"$ ($"V"$) when $\boldsymbol{O}_{2}$ or $\boldsymbol{O}_{3}$ are $\boldsymbol{h}$ ($\boldsymbol{v}$)). Hence, the intensity of the correlation is given by

$$I^{*}I\boldsymbol{=}\sum_{\boldsymbol{O}_{2}\boldsymbol{,}\boldsymbol{O}_{3}\boldsymbol{,}\boldsymbol{O}_{2}^{\boldsymbol{'}}\boldsymbol{,}\boldsymbol{O}_{3}^{\boldsymbol{'}}\boldsymbol{=}\boldsymbol{h}}^{\boldsymbol{v}} \Gamma_{\boldsymbol{O}_{3}\boldsymbol{,}\boldsymbol{O}_{2}\boldsymbol{,}\boldsymbol{O}_{2}^{\boldsymbol{'}}\boldsymbol{,}\boldsymbol{O}_{3}^{\boldsymbol{'}}}\left( \boldsymbol{e}_{3}^{*}\boldsymbol{\cdot}\boldsymbol{O}_{3} \right)\left( \boldsymbol{e}_{2}^{*}\boldsymbol{\cdot}\boldsymbol{O}_{2} \right)\left( \boldsymbol{e}_{2}\boldsymbol{\cdot}\boldsymbol{O}_{2}^{\boldsymbol{'}} \right)\left( \boldsymbol{e}_{3}\boldsymbol{\cdot}\boldsymbol{O}_{3}^{\boldsymbol{'}} \right) \left( S14 \right)$$

where

$$\Gamma_{\boldsymbol{O}_{3}\boldsymbol{,}\boldsymbol{O}_{2}\boldsymbol{,}\boldsymbol{O}_{2}^{\boldsymbol{'}}\boldsymbol{,}\boldsymbol{O}_{3}^{\boldsymbol{'}}}=\left[ \sum_{m=1}^{4} \left( \delta_{H}p_{1,m}^{H}\boldsymbol{+}\delta_{V}p_{1,m}^{V} \right)p_{2,m}^{O_{2}}p_{3,m}^{O_{3}} \right]^{*}\left[ \sum_{m^{'}=1}^{4} \left( \delta_{H}p_{1,m^{'}}^{H}\boldsymbol{+}\delta_{V}p_{1,m^{'}}^{V} \right)p_{2,m}^{O_{2}^{'}}p_{3,m}^{O_{3}^{'}} \right]\boldsymbol{=}\sum_{m,m^{'}=1}^{4} \left( \delta_{H}p_{1,m}^{H*}\boldsymbol{+}\delta_{V}p_{1,m}^{V*} \right)\left( \delta_{H}p_{1,m^{'}}^{H}\boldsymbol{+}\delta_{V}p_{1,m^{'}}^{V} \right)p_{2,m}^{O_{2}*}p_{3,m}^{O_{3}*}p_{2,m^{'}}^{O_{2}^{'}}p_{3,m^{'}}^{O_{3}^{'}}=\sum_{m,m^{'}=1}^{4} \left( p_{1,m}^{H*}p_{1,m^{'}}^{H}\boldsymbol{+}p_{1,m}^{V*}p_{1,m^{'}}^{V} \right)p_{2,m}^{O_{2}*}p_{3,m}^{O_{3}*}p_{2,m^{'}}^{O_{2}^{'}}p_{3,m^{'}}^{O_{3}^{'}}$$

$$\left( S15 \right)$$

In Eq. (S15), we apply the orthonormal relation of $\delta_{H}$ and $\delta_{V}$. For the comparison with the quantum case, we calculate the measurement of partial trace state $\mathrm{Tr}_{1}\left\{ \left| \psi_{3} \right\rangle\left\langle\psi_{3} \right| \right\}$, given by,

$$\mathrm{Tr}\left\{ \left( \left| \boldsymbol{e}_{2} \right\rangle\left| \boldsymbol{e}_{3} \right\rangle\left\langle\boldsymbol{e}_{3} \right|\left\langle\boldsymbol{e}_{2} \right| \right)\mathrm{Tr}_{1}\left\{ \left| \psi_{3} \right\rangle\left\langle\psi_{3} \right| \right\} \right\}=$$

$$\sum_{i_{2},i_{3},i_{2}^{'},i_{3}^{'}=0}^{1} \left( q_{0i_{2}i_{3}}^{*}q_{0i_{2}^{'}i_{3}^{'}}+q_{1i_{2}i_{3}}^{*}q_{1i_{2}^{'}i_{3}^{'}} \right)\langle\boldsymbol{e}_{2}\boldsymbol{e}_{3}\left| i_{2}i_{3} \right\rangle\left\langle i_{2}^{'}i_{3}^{'} \right|\boldsymbol{e}_{2}\boldsymbol{e}_{3}\rangle\left( S16 \right)$$

Notice that the computing rules of $\langle\boldsymbol{e}_{2}\boldsymbol{e}_{3}\left| i_{2}i_{3} \right\rangle\left\langle i_{2}^{'}i_{3}^{'} \right|\boldsymbol{e}_{2}\boldsymbol{e}_{3}\rangle$ and $\left( \boldsymbol{e}_{3}^{*}\boldsymbol{\cdot}\boldsymbol{O}_{3} \right)\left( \boldsymbol{e}_{2}^{*}\boldsymbol{\cdot}\boldsymbol{O}_{2} \right)\left( \boldsymbol{e}_{2}\boldsymbol{\cdot}\boldsymbol{O}_{2}^{\boldsymbol{'}} \right)\left( \boldsymbol{e}_{3}\boldsymbol{\cdot}\boldsymbol{O}_{3}^{\boldsymbol{'}} \right)$ are the same. Therefore, the key difference lies in the coefficient $\Gamma_{\boldsymbol{O}_{3}\boldsymbol{,}\boldsymbol{O}_{2}\boldsymbol{,}\boldsymbol{O}_{2}^{\boldsymbol{'}}\boldsymbol{,}\boldsymbol{O}_{3}^{\boldsymbol{'}}}$ and $q_{0i_{2}i_{3}}^{*}q_{0i_{2}^{'}i_{3}^{'}}+q_{1i_{2}i_{3}}^{*}q_{1i_{2}^{'}i_{3}^{'}}$. According to Eq. (10) in the main text, the equation set for the beam state $\left| 3E \right)\boldsymbol{=}c_{h_{1}h_{2}h_{3}}\left| \boldsymbol{h}_{1} \right)\left| \boldsymbol{h}_{2} \right)\left| \boldsymbol{h}_{3} \right)\boldsymbol{+}c_{h_{1}h_{2}v_{3}}\left| \boldsymbol{h}_{1} \right)\left| \boldsymbol{h}_{2} \right)\left| \boldsymbol{v}_{3} \right)\boldsymbol{+\ldots}\boldsymbol{+}c_{v_{1}v_{2}v_{3}}\left| \boldsymbol{v}_{1} \right)\left| \boldsymbol{v}_{2} \right)\left| \boldsymbol{v}_{3} \right)$ given by Eq. (S10) is

$$\sum_{k=1}^{4} p_{1,k}^{H}p_{2,k}^{H}p_{3,k}^{H}\boldsymbol{=}c_{h_{1}h_{2}h_{3}},\sum_{k=1}^{4} p_{1,k}^{H}p_{2,k}^{H}p_{3,k}^{V}\boldsymbol{=}c_{h_{1}h_{2}v_{3}},\ldots,\sum_{k=1}^{4} p_{1,k}^{V}p_{2,k}^{V}p_{3,k}^{V}\boldsymbol{=}c_{v_{1}v_{2}v_{3}} \left( S17 \right)$$

Define a function $S$ such that $S\left( h_{n} \right)=0$ and $S\left( v_{n} \right)=1$. Because the $c_{o_{1}o_{2}o_{3}}$ of $\left| 3E \right)$ and the $q_{i_{1}i_{2}i_{3}}$ of $\left| \psi_{3} \right\rangle$ have a one-to-one correspondence, one can simply set $c_{o_{1}o_{2}o_{3}}=q_{S\left( o_{1} \right)S\left( o_{2} \right)S\left( o_{3} \right)}$ for mimicking $\left| \psi_{3} \right\rangle$ with $\left| 3E \right)$. Then, by substituting Eq. (S17) to the coefficient $q_{0i_{2}i_{3}}^{*}q_{0i_{2}^{'}i_{3}^{'}}+q_{1i_{2}i_{3}}^{*}q_{1i_{2}^{'}i_{3}^{'}}$, one has

$$q_{0i_{2}i_{3}}^{*}q_{0i_{2}^{'}i_{3}^{'}}+q_{1i_{2}i_{3}}^{*}q_{1i_{2}^{'}i_{3}^{'}}=\left( \sum_{m=1}^{4} p_{1,m}^{H}p_{2,m}^{O_{2}}p_{3,m}^{O_{3}} \right)^{*}\left( \sum_{m=1}^{4} p_{1,m}^{H}p_{2,m}^{O_{2}^{'}}p_{3,m}^{O_{3}^{'}} \right)+\left( \sum_{m=1}^{4} p_{1,m}^{V}p_{2,m}^{O_{2}}p_{3,m}^{O_{3}} \right)^{*}\left( \sum_{m=1}^{4} p_{1,m}^{V}p_{2,m}^{O_{2}^{'}}p_{3,m}^{O_{3}^{'}} \right)=\sum_{m,m^{'}=1}^{4} \left( p_{1,m}^{H*}p_{1,m^{'}}^{H}+p_{1,m}^{V*}p_{1,m^{'}}^{V} \right)p_{2,m}^{O_{2}*}p_{3,m}^{O_{3}*}p_{2,m^{'}}^{O_{2}^{'}}p_{3,m^{'}}^{O_{3}^{'}}=\Gamma_{\boldsymbol{O}_{3}\boldsymbol{,}\boldsymbol{O}_{2}\boldsymbol{,}\boldsymbol{O}_{2}^{\boldsymbol{'}}\boldsymbol{,}\boldsymbol{O}_{3}^{\boldsymbol{'}}}$$

$$\left( S18 \right)$$

on the condition that $O_{2}=S^{-1}\left( i_{2} \right)$, $O_{3}=S^{-1}\left( i_{3} \right)$, $O_{2}^{'}=S^{-1}\left( i_{2}^{'} \right)$, and $O_{3}^{'}=S^{-1}\left( i_{3}^{'} \right)$. Hence, correlated state of $\boldsymbol{E}_{2}^{'}$ and $\boldsymbol{E}_{3}$ is equivalent to $\mathrm{Tr}_{1}\left\{ \left| \psi_{3} \right\rangle\left\langle\psi_{3} \right| \right\}$, indicating that the function of the combiner in our COCNN is equivalent to “taking the subspace of a qubit system by partial trace”. Therefore, the state space of the beams can be effectively reduced by the P-layers. Furthermore, by applying another combiner for $\boldsymbol{E}_{2}^{'}$ and $\boldsymbol{E}_{3}$, one can obtain a single beam

$$\left( \boldsymbol{E}_{2}^{'},\boldsymbol{E}_{3} \right)\underset{\to}{Combiner}$$

$$\boldsymbol{E}_{3}^{'}=\left( p_{3,1}^{H}\boldsymbol{h}+p_{3,1}^{V}\boldsymbol{v} \right)\left( \delta_{H}^{'}p_{2,1}^{H}+\delta_{V}^{'}p_{2,1}^{V} \right)\left( \delta_{H}p_{1,1}^{H}\boldsymbol{+}\delta_{V}p_{1,1}^{V} \right)f_{1}+\left( p_{3,2}^{H}\boldsymbol{h}+p_{3,2}^{V}\boldsymbol{v} \right)\left( \delta_{H}^{'}p_{2,2}^{H}+\delta_{V}^{'}p_{2,2}^{V} \right)\left( {\delta_{H}p}_{1,2}^{H}+\delta_{V}p_{1,2}^{V} \right)f_{2}+\left( p_{3,3}^{H}\boldsymbol{h}+p_{3,3}^{V}\boldsymbol{v} \right)\left( \delta_{H}^{'}p_{2,3}^{H}+\delta_{V}^{'}p_{2,3}^{V} \right)\left( \delta_{H}p_{1,3}^{H}+\delta_{V}p_{1,3}^{V} \right)f_{3}+\left( p_{3,4}^{H}\boldsymbol{h}+p_{3,4}^{V}\boldsymbol{v} \right)\left( \delta_{H}^{'}p_{2,4}^{H}+\delta_{V}^{'}p_{2,4}^{V} \right)\left( \delta_{H}p_{1,4}^{H}+\delta_{V}p_{1,4}^{V} \right)f_{4}$$

$$\left( S19 \right)$$

Here, $\delta_{H}^{'}$ and $\delta_{V}^{'}$ also have the relation $\delta_{H}^{'}\delta_{V}^{'}=0$ and $\delta_{H}^{'}\delta_{H}^{'}=\delta_{V}^{'}\delta_{V}^{'}=1$. However, $\delta_{H}^{'}$ and $\delta_{V}^{'}$ do not interact with $\delta_{H}$ and $\delta_{V}$, indicating that the action introducing $\delta_{H}^{'}$ and $\delta_{V}^{'}$ is independent of the action introducing $\delta_{H}$ and $\delta_{V}$ . This can be easily implemented by shifting the phase of $\boldsymbol{E}_{1}^{\boldsymbol{'}}$ by a random number independent of that applied to $\boldsymbol{E}_{2}$. Similar to the calculation by Eq. (S13) and Eq. (S14), the measurement on $\boldsymbol{E}_{3}^{'}$gives $I_{3}^{*}I_{3}$, where

$$I_{3}=\int\left( {\boldsymbol{e}_{3}\boldsymbol{\cdot}\boldsymbol{E}}_{3}^{'} \right)d\Omega=\boldsymbol{e}_{3}\boldsymbol{\cdot}\sum_{m=1}^{4} \left( \delta_{H}^{'}p_{2,m}^{H}+\delta_{V}^{'}p_{2,m}^{V} \right)\left( \delta_{H}p_{1,m}^{H}\boldsymbol{+}\delta_{V}p_{1,m}^{V} \right)\left( p_{3,m}^{H}\boldsymbol{h}+p_{3,m}^{V}\boldsymbol{v} \right)=\sum_{m=1}^{4} \left( \delta_{H}^{'}p_{2,m}^{H}+\delta_{V}^{'}p_{2,m}^{V} \right)\left( \delta_{H}p_{1,m}^{H}\boldsymbol{+}\delta_{V}p_{1,m}^{V} \right)p_{3,m}^{H}\left( \boldsymbol{e}_{3}\boldsymbol{\cdot}\boldsymbol{h} \right)+\sum_{m=1}^{4} \left( \delta_{H}^{'}p_{2,m}^{H}+\delta_{V}^{'}p_{2,m}^{V} \right)\left( \delta_{H}p_{1,m}^{H}\boldsymbol{+}\delta_{V}p_{1,m}^{V} \right)p_{3,m}^{V}\left( \boldsymbol{e}_{3}\boldsymbol{\cdot}\boldsymbol{v} \right)$$

$$\left( S20 \right)$$

Then,

$$I_{3}^{*}I_{3}=\left[ \sum_{m=1}^{4} \left( \delta_{H}^{'}p_{2,m}^{H}+\delta_{V}^{'}p_{2,m}^{V} \right)\left( \delta_{H}p_{1,m}^{H}\boldsymbol{+}\delta_{V}p_{1,m}^{V} \right)p_{3,m}^{H}\left( \boldsymbol{e}_{3}\boldsymbol{\cdot}\boldsymbol{h} \right)+\sum_{m=1}^{4} \left( \delta_{H}^{'}p_{2,m}^{H}+\delta_{V}^{'}p_{2,m}^{V} \right)\left( \delta_{H}p_{1,m}^{H}\boldsymbol{+}\delta_{V}p_{1,m}^{V} \right)p_{3,m}^{V}\left( \boldsymbol{e}_{3}\boldsymbol{\cdot}\boldsymbol{v} \right) \right]^{*}\left[ \sum_{m=1}^{4} \left( \delta_{H}^{'}p_{2,m}^{H}+\delta_{V}^{'}p_{2,m}^{V} \right)\left( \delta_{H}p_{1,m}^{H}\boldsymbol{+}\delta_{V}p_{1,m}^{V} \right)p_{3,m}^{H}\left( \boldsymbol{e}_{3}\boldsymbol{\cdot}\boldsymbol{h} \right)+\sum_{m=1}^{4} \left( \delta_{H}^{'}p_{2,m}^{H}+\delta_{V}^{'}p_{2,m}^{V} \right)\left( \delta_{H}p_{1,m}^{H}\boldsymbol{+}\delta_{V}p_{1,m}^{V} \right)p_{3,m}^{V}\left( \boldsymbol{e}_{3}\boldsymbol{\cdot}\boldsymbol{v} \right) \right]$$

$$=\sum_{\boldsymbol{O}_{3}\boldsymbol{,}\boldsymbol{O}_{3}^{\boldsymbol{'}}\boldsymbol{=h}}^{\boldsymbol{v}} \Gamma_{\boldsymbol{O}_{3}\boldsymbol{,}\boldsymbol{O}_{3}^{\boldsymbol{'}}}^{'}\left( \boldsymbol{e}_{3}^{*}\boldsymbol{\cdot}\boldsymbol{O}_{3} \right)\left( \boldsymbol{e}_{3}\boldsymbol{\cdot}\boldsymbol{O}_{3}^{\boldsymbol{'}} \right)$$

$$\left( S21 \right)$$

in which,

$$\Gamma_{\boldsymbol{O}_{3}\boldsymbol{,}\boldsymbol{O}_{3}^{\boldsymbol{'}}}^{'}=\left[ \sum_{m=1}^{4} \left( \delta_{H}^{'}p_{2,m}^{H}+\delta_{V}^{'}p_{2,m}^{V} \right)\left( \delta_{H}p_{1,m}^{H}\boldsymbol{+}\delta_{V}p_{1,m}^{V} \right)p_{3,m}^{O_{3}} \right]^{\boldsymbol{*}}\left[ \sum_{m=1}^{4} \left( \delta_{H}^{'}p_{2,m}^{H}+\delta_{V}^{'}p_{2,m}^{V} \right)\left( \delta_{H}p_{1,m}^{H}\boldsymbol{+}\delta_{V}p_{1,m}^{V} \right)p_{3,m}^{O_{3}^{'}} \right]=\sum_{m,m^{'}=1}^{4} \left( p_{2,m}^{H*}p_{1,m}^{H*}p_{2,m^{'}}^{H}p_{1,m^{'}}^{H}+p_{2,m}^{H*}p_{1,m}^{V*}p_{2,m^{'}}^{H}p_{1,m^{'}}^{V}+p_{2,m}^{V*}p_{1,m}^{H*}p_{2,m^{'}}^{V}p_{1,m^{'}}^{H}+p_{2,m}^{V*}p_{1,m}^{V*}p_{2,m^{'}}^{V}p_{1,m^{'}}^{V} \right)p_{3,m}^{O_{3}*}p_{3,m^{'}}^{O_{3}^{'}}$$

$$\left( S22 \right)$$

Also, for the comparison with the quantum case, we calculate the measurement on $\mathrm{Tr}_{1,2}\left\{ \left| \psi_{3} \right\rangle\left\langle\psi_{3} \right| \right\}$ given by,

$$\mathrm{Tr}\left\{ \left| \boldsymbol{e}_{3} \right\rangle\left\langle\boldsymbol{e}_{3} \right|Tr_{1,2}\left\{ \left| \psi_{3} \right\rangle\left\langle\psi_{3} \right| \right\} \right\}=\sum_{i_{3},i_{3}^{'}=0}^{1} \left( q_{00i_{3}}^{*}q_{00i_{3}^{'}}+q_{10i_{3}}^{*}c_{10i_{3}^{'}}+q_{01i_{3}}^{*}q_{01i_{3}^{'}}+q_{11i_{3}}^{*}q_{11i_{3}^{'}} \right)\left\langle\boldsymbol{e}_{3}\left| i_{3} \right\rangle\left\langle i_{3}^{'} \right|\boldsymbol{e}_{3} \right\rangle$$

$$\left( S23 \right)$$

By using the same trick for obtaining Eq. (S18), one has

$$q_{00i_{3}}^{*}q_{00i_{3}^{'}}+q_{10i_{3}}^{*}c_{10i_{3}^{'}}+q_{01i_{3}}^{*}q_{01i_{3}^{'}}+q_{11i_{3}}^{*}q_{11i_{3}^{'}}=\sum_{m,m^{'}=0}^{1} \left( p_{1,m}^{H*}p_{2,m}^{H*}p_{1,m^{'}}^{H}p_{2,m^{'}}^{H}+p_{1,m}^{V*}p_{2,m}^{H*}p_{1,m^{'}}^{V}p_{2,m^{'}}^{H}+p_{1,m}^{H*}p_{2,m}^{V*}p_{1,m^{'}}^{H}p_{2,m^{'}}^{V}+p_{1,m}^{V*}p_{2,m}^{V*}p_{1,m^{'}}^{V}p_{2,m^{'}}^{V} \right)p_{3,m}^{O_{3}*}p_{3,m^{'}}^{O_{3}^{'}}=\Gamma_{\boldsymbol{O}_{3}\boldsymbol{,}\boldsymbol{O}_{3}^{\boldsymbol{'}}}^{'}$$

$$\left( S24 \right)$$

on the condition that $O_{3}=S^{-1}\left( i_{3} \right)$ and $O_{3}^{'}=S^{-1}\left( i_{3}^{'} \right)$. More generally, consider the $N$ beams $\boldsymbol{E}_{k}=\sum_{m=1}^{M} f_{m}\left( p_{k,m}^{H}\boldsymbol{h}+p_{k,m}^{V}\boldsymbol{v} \right)$, $k=1,\ldots,N$, for mimicking the $N$*-*qubit state,

$$\left| \psi_{N} \right\rangle=\sum_{i_{1},i_{2},\ldots, i_{N}=0}^{1} q_{i_{1}i_{2}\ldots i_{N}}\left| i_{1} \right\rangle\left| i_{2} \right\rangle\ldots\left| i_{N} \right\rangle\left( S25 \right)$$

The constrains for their relation are

$$\sum_{k=1}^{M} p_{1,k}^{H}\boldsymbol{\ldots}p_{N,k}^{H}\boldsymbol{=}q_{0\ldots0},\sum_{k=1}^{M} p_{1,k}^{H}\boldsymbol{\ldots}p_{N,k}^{V}\boldsymbol{=}q_{0\ldots1},\ldots,\sum_{k=1}^{M} p_{1,k}^{V}\boldsymbol{\ldots}p_{N,k}^{V}\boldsymbol{=}q_{1\ldots1} \left( S26 \right)$$

The reduced density matrix of the second to last qubits of $\left| \psi_{N} \right\rangle$ can be obtained by

$$\mathrm{Tr}_{1}\left\{ \left| \psi_{N} \right\rangle\left\langle\psi_{N} \right| \right\}=\left( \left\langle0 \right|_{1}\otimes I^{\otimes\left( N-1 \right)} \right)\left| \psi_{N} \right\rangle\left\langle\psi_{N} \right|\left( \left| 0 \right\rangle_{1}\otimes I^{\otimes\left( N-1 \right)} \right)+\left( \left\langle1 \right|_{1}\otimes I^{\otimes\left( N-1 \right)} \right)\left| \psi_{N} \right\rangle\left\langle\psi_{N} \right|\left( \left| 1 \right\rangle_{1}\otimes I^{\otimes\left( N-1 \right)} \right)=\sum_{i_{2},\ldots,i_{N},i_{2}^{'},\ldots,i_{3}^{'}=0}^{1} \left( q_{0i_{2}i_{3}\ldots i_{N}}^{*}q_{0i_{2}^{'}i_{3}^{'}\ldots i_{N}^{'}}+q_{1i_{2}i_{3}\ldots i_{N}}^{*}q_{1i_{2}^{'}i_{3}^{'}\ldots i_{N}^{'}} \right)\left| i_{2}i_{3}\ldots i_{N} \right\rangle\left\langle i_{2}^{'}i_{3}^{'}\ldots i_{N}^{'} \right|$$

$$\left( S27 \right)$$

By using the combiner, one has $\left( \boldsymbol{E}_{1},\boldsymbol{E}_{2},\ldots,\boldsymbol{E}_{N} \right)\underset{\to}{Combiner}\left( \boldsymbol{E}_{2}^{\boldsymbol{'}},\ldots,\boldsymbol{E}_{N} \right)$. In the similar manner, by calculating the coefficients $\Gamma_{\boldsymbol{O}_{N}\boldsymbol{\ldots O,}\boldsymbol{O}_{2}^{\boldsymbol{'}}\boldsymbol{\ldots}\boldsymbol{O}_{N}^{\boldsymbol{'}}}$ in the intensity of the correlation $\int\left( \boldsymbol{e}_{2}\boldsymbol{\cdot}\boldsymbol{E}_{2}^{\boldsymbol{'}} \right)\ldots\left( \boldsymbol{e}_{N}\boldsymbol{\cdot}\boldsymbol{E}_{N} \right)d\Omega$, the following relation can be given,

$$q_{0i_{2}i_{3}\ldots i_{N}}^{*}q_{0i_{2}^{'}i_{3}^{'}\ldots i_{N}^{'}}+q_{1i_{2}i_{3}\ldots i_{N}}^{*}q_{1i_{2}^{'}i_{3}^{'}\ldots i_{N}^{'}}=\sum_{m,m^{'}=1}^{M} \left( p_{1,m}^{H*}p_{1,m^{'}}^{H}+p_{1,m}^{V*}p_{1,m^{'}}^{V} \right)p_{2,m}^{O_{2}*}\ldots p_{N,m}^{O_{N}*}p_{2,m^{'}}^{O_{2}^{'}}\ldots p_{N,m^{'}}^{O_{N}^{'}}=\Gamma_{\boldsymbol{O}_{N}\boldsymbol{\ldots}\boldsymbol{O}\boldsymbol{,}\boldsymbol{O}_{2}^{\boldsymbol{'}}\boldsymbol{\ldots}\boldsymbol{O}_{N}^{\boldsymbol{'}}}$$

$$\left( S28 \right)$$

**Table S1** The expressions of the beams after each combiner.

| Number | Notations | Expression |
| --- | --- | --- |
| $N$ | $\boldsymbol{E}_{1}\boldsymbol{,}\boldsymbol{E}_{2}\boldsymbol{,}\boldsymbol{E}_{3}\boldsymbol{,\ldots,}\boldsymbol{E}_{N}$ | $\boldsymbol{E}_{k}=\sum_{m=1}^{M} f_{m}\left( p_{k,m}^{H}\boldsymbol{h}+p_{k,m}^{V}\boldsymbol{v} \right)$  $k=1,\ldots,N$ |
| $N-1$ | $\boldsymbol{E}_{2}^{\boldsymbol{'}}\boldsymbol{,}\boldsymbol{E}_{3}\boldsymbol{,\ldots,}\boldsymbol{E}_{N}$ | $\begin{matrix} \boldsymbol{E}_{2}=\sum_{m=1}^{M} f_{m}\left( \delta_{H}p_{1,1}^{H}\boldsymbol{+}\delta_{V}p_{1,1}^{V} \right)\left( p_{2,m}^{H}\boldsymbol{h}+p_{2,m}^{V}\boldsymbol{v} \right) \\ \boldsymbol{E}_{k}=\sum_{m=1}^{M} f_{m}\left( p_{k,m}^{H}\boldsymbol{h}+p_{k,m}^{V}\boldsymbol{v} \right), k=3,\ldots,N \end{matrix}$ |
| $N-2$ | $\boldsymbol{E}_{3}^{\boldsymbol{'}}\boldsymbol{,\ldots,}\boldsymbol{E}_{N}$ | $\begin{matrix} \boldsymbol{E}_{3}=\sum_{m=1}^{M} f_{m}\left( \delta_{H}^{'}p_{2,1}^{H}\boldsymbol{+}\delta_{V}^{'}p_{2,1}^{V} \right)\left( \delta_{H}p_{1,1}^{H}\boldsymbol{+}\delta_{V}p_{1,1}^{V} \right)\left( p_{2,m}^{H}\boldsymbol{h}+p_{2,m}^{V}\boldsymbol{v} \right) \\ \boldsymbol{E}_{k}=\sum_{m=1}^{M} f_{m}\left( p_{k,m}^{H}\boldsymbol{h}+p_{k,m}^{V}\boldsymbol{v} \right), k=4,\ldots,N \end{matrix}$ |
| $\boldsymbol{\vdots}$ | $\boldsymbol{\vdots}$ | $\boldsymbol{\vdots}$ |
| $1$ | $\boldsymbol{E}_{N}^{\boldsymbol{'}}$ | $\boldsymbol{E}_{N}=\sum_{m=1}^{M} f_{m}\left[ \prod_{k=1}^{N} \left( \delta_{H}^{(k-1)}p_{k,1}^{H}\boldsymbol{+}\delta_{V}^{(k-1)}p_{k,1}^{V} \right) \right]\left( p_{2,m}^{H}\boldsymbol{h}+p_{2,m}^{V}\boldsymbol{v} \right)$ |

The sequential reduction in the state space of the beams by multiple combiners can be finally given by Table S1. In the table, $\delta_{H}^{(0)}=\delta_{H},\delta_{H}^{(1)}=\delta_{H}^{'}, \delta_{H}^{(2)}=\delta_{H}^{''},\ldots,\delta_{H}^{\left( N-1 \right)}=\delta_{H}^{''\ldots'}$, and the same goes for $\delta_{V}^{(k)}$. Using such a notation, one could conclude that the last beam $\boldsymbol{E}_{N}^{\boldsymbol{'}}$ in the procedure contains the information of other beams. The resultant polarization components of the beam $\boldsymbol{E}_{N}^{\boldsymbol{'}}$ are basically the products of the polarization components of other beams. Such a procedure only requires $N-1$ steps. Also, we would like to mention that there are other potential methods for implementing the above operation. For example, in the subspace when $q_{1,m}^{H}q_{1,m'}^{H}+q_{1,m}^{V}q_{1,m'}^{V}=1$, the state encoded by $\boldsymbol{E}_{2}\boldsymbol{,\ldots,}\boldsymbol{E}_{N}$ is naturally equivalent to the $\mathrm{Tr}_{1}\left\{ \left| \psi_{N} \right\rangle\left\langle\psi_{N} \right| \right\}$, and no non-linear operation is required. If the number of the constrains is sufficient, such a subspace can be found. A simple solution is to require $q_{1,1}^{H}=q_{1,2}^{H}=\ldots=q_{1,N}^{H}$ and $q_{1,1}^{V}=q_{1,2}^{V}=\ldots=q_{1,N}^{V}$, meaning that the polarizations of all the states are the same. This also means that such a beam is equivalent to a single mode polarized beam. However, to implement $q_{1,1}^{H}=q_{1,2}^{H}=\ldots=q_{1,N}^{H}$ and $q_{1,1}^{V}=q_{1,2}^{V}=\ldots=q_{1,N}^{V}$ while keeping the constrains Eq. (S26) might be tricky.

**S3. The connection between COCNNs and QCNNs.**

We discuss the connections between the COCNNs and QCNNs  [3] in details. A QCNN is basically a combination of unitary operators and the measurement-based operators. The data to be processed is encoded by quantum states. By applying the layer of unitary operators, the data-encoded state can be transformed to the feature-encoded states. Then, the space of the feature-encoded state is suppressed by measurement-based gate. In fact, the function of each block in the QCNN scheme can be correspondingly performed in our COCNN scheme. It is a one-to-one correspondence.


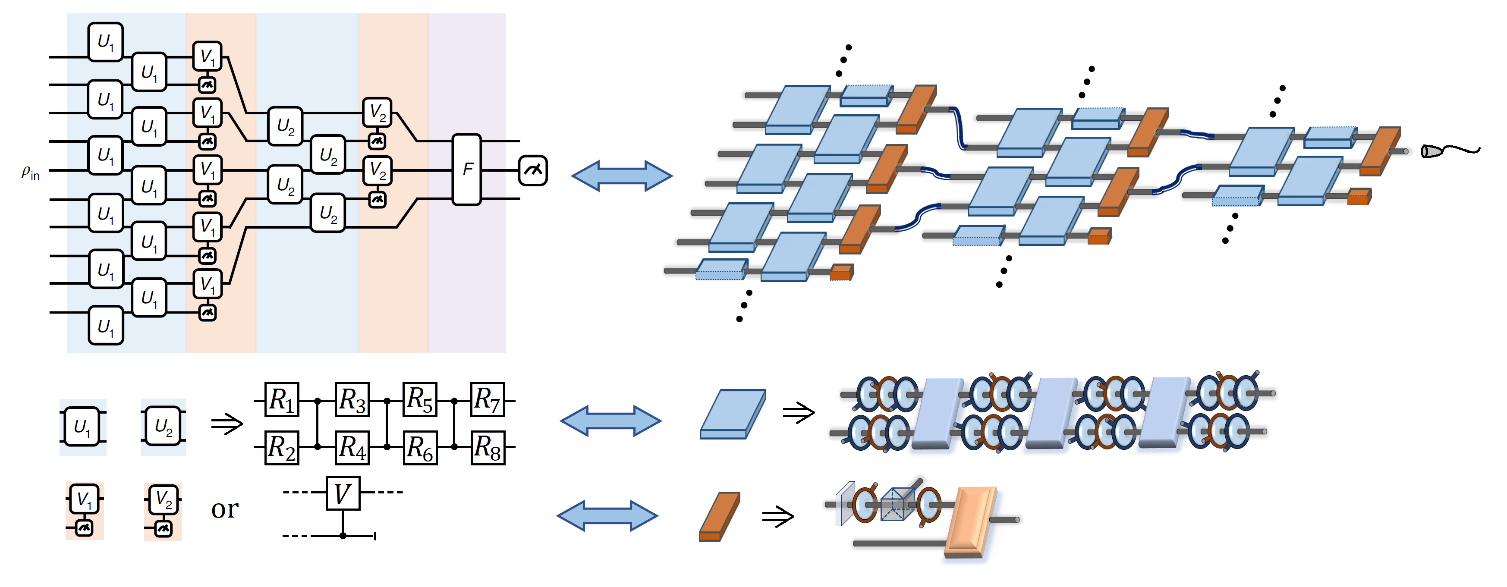


**Figure S1** The relation between the QCNN (left) and the COCNN (right). The upper panel is the correspondence between the whole networks. The middle panel gives the correspondence between the 2-qubit gates ($U_{1}$ and $U_{2}$) and the 2-beam operation (the specific description is given in the main text). The bottom panel gives the correspondence between the measurement-based gate ($V_{1}$ and $V_{2}$ associated with a measurement) and the combiner (the specific description is also given in the main text).

We firstly discuss the part of the convolution. As shown in the left of Fig. S1, the layer of the quantum convolution in a QCNN (blue area) is composed of 2-qubit gates. According to the theory of quantum computing, the unitary gate on two qubits can be decomposed into 8 single-qubit gates and 3 CNOT gates. Because the CNOT gates and CZ gates can be transformed into each other, one can change the decomposition into 8 single qubit gates with 3 CZ gates, as shown by the middle of the left Fig. S1. The 2-qubit gates $U_{1}$ and $U_{2}$ in the middle of the left Fig. S1, or more generally, an arbitrary 2-qubit gate $U_{2q}$ is expressed by

$$U_{2q}=\left( U_{R1}\otimes U_{R2} \right)U_{\mathrm{CZ}}\left( U_{R3}\otimes U_{R4} \right)U_{\mathrm{CZ}}\left( U_{R5}\otimes U_{R6} \right)U_{\mathrm{CPhase}}\left( U_{R7}\otimes U_{R8} \right)\text{ }\text{ }\left( S29 \right)$$

where $U_{R1}$ to $U_{R8}$ are the matrices of the single qubit gates. The relation of the CZ gate and the CNOT gate is given by,

$$U_{\mathrm{CNOT}}=\left( I\otimes U_{H} \right)U_{\mathrm{CZ}}\left( I\otimes U_{H} \right) \left( S30 \right)$$

where

$$U_{\mathrm{CNOT}}=\left( \begin{matrix} 1 & 0 & 0 & 0 \\ 0 & 1 & 0 & 0 \\ 0 & 0 & 0 & 1 \\ 0 & 0 & 1 & 0 \end{matrix} \right), U_{\mathrm{CZ}}=\left( \begin{matrix} 1 & 0 & 0 & 0 \\ 0 & 1 & 0 & 0 \\ 0 & 0 & 1 & 0 \\ 0 & 0 & 0 & -1 \end{matrix} \right) \left( S31 \right)$$

According to Eq. (S6) in S1, the OMU on the beam state $\left| 2E \right)$ can also be described by the same matrix with $U_{CZ}$ in the basis $\left\{ \left| \boldsymbol{h}_{1} \right)\left| \boldsymbol{h}_{2} \right)\boldsymbol{,}\left| \boldsymbol{h}_{1} \right)\left| \boldsymbol{v}_{2} \right)\boldsymbol{,}\left| \boldsymbol{v}_{1} \right)\left| \boldsymbol{h}_{2} \right)\boldsymbol{,}\left| \boldsymbol{v}_{1} \right)\left| \boldsymbol{v}_{2} \right) \right\}$. By using a Q-H-Q, a unitary transformation $U_{R}$ can be given by [4],

$$U_{R}\left( \xi,\eta,\zeta\right)=J_{Q}\left( \frac{\pi}{4}+\frac{\xi}{2} \right)\cdot J_{H}\left( -\frac{\pi}{4}+\frac{\xi+\eta-\zeta}{4} \right)\cdot J_{Q}\left( \frac{\pi}{4}-\frac{\zeta}{2} \right) \left( S32 \right)$$

where the definition of $U_{R}$ is given by,

$$U_{R}\left( \xi,\eta,\zeta\right)\equiv\exp\left( -i\frac{1}{2}\xi Y \right)\exp\left( -i\frac{1}{2}\eta Z \right)\exp\left( -i\frac{1}{2}\zeta Y \right) \text{(S33)}$$

The Jones matrices of an HWP and a QWP (the horizontal and vertical polarization states as basis) are given by

$$J_{H}\left( \varphi\right)=\left( \begin{matrix} -i\cos2\varphi& -i\sin2\varphi\\ -i\sin2\varphi& i\cos2\varphi\end{matrix} \right), J_{Q}\left( \varphi\right)=\frac{1}{\sqrt{2}}\left( \begin{matrix} 1-i\cos2\varphi& -i\sin2\varphi\\ -i\sin2\varphi& 1+i\cos2\varphi\end{matrix} \right) \text{(}\text{S34}\text{)}$$

where $\varphi$ is the orientation angle of the fast axis of the HWP or QWP. Therefore, the 2-beam operation $U_{2E}$ in the main text by using 8 Q-H-Qs and 3 OMUs (blue box in Fig. 1 of the main text, or the middle of the right Fig. S1) can be expressed by the same matrix with Eq. (S29). Also, according to our discussion of the operations in $N$-beam case, the network of the $U_{2E}$s, which is called a convolutional layer (C-layer) can perform the same function of the network of $U_{2q}$s, which is the quantum convolutional layer given by QCNN.

We secondly discuss the measurement-based gate. In Ref. [3], the function of the measurement-based gate is to apply a single qubit rotation gate (such as $V_{1}$ and $V_{2}$ in the bottom of the left Fig. S1) if the measurement of the adjacent qubit shows that it is in the state $\left| + \right\rangle=\left( \left| 0 \right\rangle+\left| 1 \right\rangle\right)/\sqrt{2}$, and to do nothing otherwise. Such a gate is equivalent to the setup that firstly applies a two-qubit control rotation gate, and then simply leaves the control qubit away. This can be seen by the followings. According to the definition, if the single qubit gate is the denoted by $V$ and the orthonormal single qubit state of $\left| + \right\rangle$ is denotated by $\left| - \right\rangle$, measurement-based gate on a 2-qubit state $\left| \psi_{2} \right\rangle$ is expressed by

$$V^{\dagger}Tr_{1}\left\{ \left( \left| + \right\rangle\left\langle+ \right|\otimes I \right)\left| \psi_{2} \right\rangle\left\langle\psi_{2} \right| \right\}V+Tr_{1}\left\{ \left( \left| - \right\rangle\left\langle- \right|\otimes I \right)\left| \psi_{2} \right\rangle\left\langle\psi_{2} \right| \right\} \text{(}\text{S35}\text{)}$$

Like in the main text, $Tr_{1}$ denotes the partial trace of the first qubit state. By using the definition of $Tr_{1}$,

$$Tr_{1}\left\{ \left| \psi_{2} \right\rangle\left\langle\psi_{2} \right| \right\}=\left( \left\langle+ \right|\otimes I \right)\left( \left| \psi_{2} \right\rangle\left\langle\psi_{2} \right| \right)\left( \left| + \right\rangle\otimes I \right)+\left( \left\langle- \right|\otimes I \right)\left( \left| \psi_{2} \right\rangle\left\langle\psi_{2} \right| \right)\left( \left| - \right\rangle\otimes I \right) \text{(}\text{S36}\text{)}$$

and the identity $I=\left| + \right\rangle\left\langle+ \right|+\left| - \right\rangle\left\langle- \right|$, one can further calculate Eq. (S35) and obtain,

$$V^{\dagger}\left( \left\langle+ \right|\otimes I \right)\left( \left| \psi_{2} \right\rangle\left\langle\psi_{2} \right| \right)\left( \left| + \right\rangle\otimes I \right)V+\left( \left\langle- \right|\otimes I \right)\left( \left| \psi_{2} \right\rangle\left\langle\psi_{2} \right| \right)\left( \left| - \right\rangle\otimes I \right)$$

$$=\left( \left\langle+ \right|\otimes V^{\dagger} \right)\left( \left| \psi_{2} \right\rangle\left\langle\psi_{2} \right| \right)\left( \left| + \right\rangle\otimes V \right)+\left( \left\langle- \right|\otimes I \right)\left( \left| \psi_{2} \right\rangle\left\langle\psi_{2} \right| \right)\left( \left| - \right\rangle\otimes I \right)\text{ }\text{(}\text{S37}\text{)}$$

Eq. (S37) is because $V$ only operate on the second qubit.

In another way of the performing the measurement-based gate, one can firstly apply the control rotation gate in the basis of $\left\{ \left| + \right\rangle,\left| - \right\rangle\right\}$, and then to look into the space of the target qubit. For comparison with the above, the rotation of the target qubit is also denoted by $V$. Hence, the mathematical description can be given by

$$Tr_{1}\left\{ \left( \left| + \right\rangle\left\langle+ \right|\otimes V^{\dagger}+\left| - \right\rangle\left\langle- \right|\otimes I \right)\left| \psi_{2} \right\rangle\left\langle\psi_{2} \right|\left( \left| + \right\rangle\left\langle+ \right|\otimes V+\left| - \right\rangle\left\langle- \right|\otimes I \right) \right\}=Tr_{1}\left\{ \left( \left| + \right\rangle\left\langle+ \right|\otimes V^{\dagger} \right)\left| \psi_{2} \right\rangle\left\langle\psi_{2} \right|\left( \left| + \right\rangle\left\langle+ \right|\otimes V \right) \right\}+Tr_{1}\left\{ \left( \left| + \right\rangle\left\langle+ \right|\otimes V^{\dagger} \right)\left| \psi_{2} \right\rangle\left\langle\psi_{2} \right|\left( \left| - \right\rangle\left\langle- \right|\otimes I \right) \right\}+Tr_{1}\left\{ \left( \left| - \right\rangle\left\langle- \right|\otimes I \right)\left| \psi_{2} \right\rangle\left\langle\psi_{2} \right|\left( \left| + \right\rangle\left\langle+ \right|\otimes V \right) \right\}+Tr_{1}\left\{ \left( \left| - \right\rangle\left\langle- \right|\otimes I \right)\left| \psi_{2} \right\rangle\left\langle\psi_{2} \right|\left( \left| - \right\rangle\left\langle- \right|\otimes I \right) \right\}$$

$$\text{ }\text{(}\text{S38}\text{)}$$

It is easy to check that each term in Eq. (S38) satisfies,

$$Tr_{1}\left\{ \left( \left| + \right\rangle\left\langle+ \right|\otimes V^{\dagger} \right)\left| \psi_{2} \right\rangle\left\langle\psi_{2} \right|\left( \left| + \right\rangle\left\langle+ \right|\otimes V \right) \right\}=\left( \left\langle+ \right|\otimes V^{\dagger} \right)\left| \psi_{2} \right\rangle\left\langle\psi_{2} \right|\left( \left| + \right\rangle\otimes V \right)$$

$$Tr_{1}\left\{ \left( \left| + \right\rangle\left\langle+ \right|\otimes V^{\dagger} \right)\left| \psi_{2} \right\rangle\left\langle\psi_{2} \right|\left( \left| - \right\rangle\left\langle- \right|\otimes I \right) \right\}=0$$

$$Tr_{1}\left\{ \left( \left| - \right\rangle\left\langle- \right|\otimes I \right)\left| \psi_{2} \right\rangle\left\langle\psi_{2} \right|\left( \left| + \right\rangle\left\langle+ \right|\otimes V \right) \right\}=0$$

$$Tr_{1}\left\{ \left( \left| - \right\rangle\left\langle- \right|\otimes I \right)\left| \psi_{2} \right\rangle\left\langle\psi_{2} \right|\left( \left| - \right\rangle\left\langle- \right|\otimes I \right) \right\}=\left( \left\langle- \right|\otimes I \right)\left| \psi_{2} \right\rangle\left\langle\psi_{2} \right|\left( \left| - \right\rangle\otimes I \right) \text{ }\text{(}\text{S39}\text{)}$$

By subscripting Eq. (S39) to Eq. (S38), one can find that Eq. (S38) equals to Eq. (S37).

In summary, the measurement-based gate can be given by firstly applying the control rotation gate and then looking into the space of the target qubit. The corresponding procedure of “looking into the space of the target qubit” implemented by the combiner as we specified by section S2. The control gate like $\left| + \right\rangle\left\langle+ \right|\otimes V+\left| - \right\rangle\left\langle- \right|\otimes I$ can also be correspondingly implemented by the 2-beam operation. The specific strategy can be given as follows. By using the ABC-decomposition [1], one has,

$$\left| 0 \right\rangle\left\langle0 \right|\otimes I+\left| 1 \right\rangle\left\langle1 \right|\otimes V=\left( I\otimes C \right)U_{\mathrm{CNOT}}\left( I\otimes B \right)U_{\mathrm{CNOT}}\left( R_{\alpha}\otimes A \right) \text{(}\text{S40}\text{)}$$

where $A$, $B$, and $C$ are single qubit unitary gates, and $R_{\alpha}$ is the phase shift gate. For a given $V$,

$$V=e^{i\alpha}R_{Z}\left( \theta_{1} \right)R_{Y}\left( \theta_{2} \right)R_{Z}\left( \theta_{3} \right) \text{(}\text{S41}\text{)}$$

the expression of $A$, $B$, and $C$ are

$$A=R_{Z}\left( \theta_{1} \right)R_{Y}\left( \frac{\theta_{2}}{2} \right)$$

$$B=R_{Y}\left( -\frac{\theta_{2}}{2} \right)R_{Z}\left( -\frac{\theta_{3}}{2}-\frac{\theta_{1}}{2} \right)$$

$$C=R_{Z}\left( \frac{\theta_{3}}{2}-\frac{\theta_{1}}{2} \right)\text{ }\text{(}\text{S42}\text{)}$$

$R_{Y}$ and $R_{Z}$ are Pauli-Y and -Z rotations. Notice that the analogy of each term in Eq. (S40) for the beam state has been discussed, including the Q-H-Qs analog to the single qubit rotations, and the OMU with Q-H-Qs analog to the $U_{\mathrm{CNOT}}$. Consider Eq. (S32) - (S34), the Pauli-*Y* and -*Z* rotations can be implemented by

$$R_{Y}\left( \xi\right)\to J_{Q}\left( \frac{\pi}{4}+\frac{\xi}{2} \right)\cdot J_{H}\left( -\frac{\pi}{4}+\frac{\xi}{4} \right)\cdot J_{Q}\left( \frac{\pi}{4} \right)$$

$$R_{Z}\left( \eta\right)\to J_{Q}\left( \frac{\pi}{4} \right)\cdot J_{H}\left( -\frac{\pi}{4}+\frac{\eta}{4} \right)\cdot J_{Q}\left( \frac{\pi}{4} \right)\text{ }\text{(}\text{S43}\text{)}$$

Furthermore, the (combination of) Q-H-Qs that implements $A$, $B$, and $C$ can be given by,

$$A\to J_{Q}\left( \frac{\pi}{4} \right)\cdot J_{H}\left( -\frac{\pi}{4}+\frac{\theta_{1}}{4} \right)\cdot J_{Q}\left( \frac{\pi}{4} \right)J_{Q}\left( \frac{\pi}{4}+\frac{\theta_{2}}{4} \right)\cdot J_{H}\left( -\frac{\pi}{4}+\frac{\theta_{2}}{8} \right)\cdot J_{Q}\left( \frac{\pi}{4} \right)$$

$$B\to J_{Q}\left( \frac{\pi}{4}-\frac{\theta_{2}}{4} \right)\cdot J_{H}\left( -\frac{\pi}{4}-\frac{\theta_{2}}{8} \right)\cdot J_{Q}\left( \frac{\pi}{4} \right)J_{Q}\left( \frac{\pi}{4} \right)\cdot J_{H}\left( -\frac{\pi}{4}-\frac{\theta_{3}}{4}-\frac{\theta_{1}}{4} \right)\cdot J_{Q}\left( \frac{\pi}{4} \right)$$

$$C\to J_{Q}\left( \frac{\pi}{4} \right)\cdot J_{H}\left( -\frac{\pi}{4}+\frac{\theta_{3}}{4}-\frac{\theta_{1}}{4} \right)\cdot J_{Q}\left( \frac{\pi}{4} \right)\text{ }\text{(}\text{S44}\text{)}$$

By Eqs. (S43), we only show that the function of the gates in a QCNN can be correspondingly implemented in a COCNN. The sequence of the HWPs and the QWPs is not optimal.

Considering that the function of the basic blocks in a QCNN can be accordingly realized in the scheme of the COCNN, the processing of beam states by the COCNN is equivalent to the manipulation of the qubits by the QCNN.

For the specific QCNN for phase recognition, we briefly analyze the specific implementation of the networks as follows. There are several quantum gates are required. According to the circuit in Ref. [3], the gates are CZ gates, SWAP gates, Toffoli gates, and measurement-based gates. The setup for the analogy of a CZ gate is discussed above. The function of a SWAP gate can be realized by simply changing the position of the beams. The measurement-based gates here manipulate the qubit as a Pauli-*Z* gate if its adjacent qubit is found to be in state $\left| + \right\rangle$, and do nothing otherwise. Based on the above discussion, the function of this gate can be realized by firstly rotating the one beam basis from $\left\{ \left| \boldsymbol{h} \right),\left| \boldsymbol{v} \right) \right\}$ to $\left\{ \left( \left| \boldsymbol{h} \right)\pm\left| \boldsymbol{v} \right) \right)/\sqrt{2} \right\}$, then applying the OMU, and lastly applying the combiner for the beam with another so that only one beam is left. To realize the function of a Toffoli gate, one can consider the decomposition shown by Fig. S2 [1]. The gate $T$ is defined by $T=\exp\left( i\pi Z/8 \right)$, whose function can be realized by a Q-H-Q $J_{Q}\left( \pi/4 \right)\cdot J_{H}\left( -5\pi/16 \right)\cdot J_{Q}\left( \pi/4 \right)$. The function of a Hadamard gate can be realized by an HWP oriented at $\pi/8$. Therefore, it is also implementable in the scheme of the COCNN, similar to the trick in the above discussion.


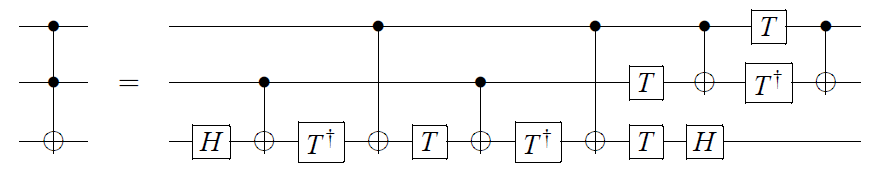


**Figure S2** The decomposition of a Toffoli gate by using $T$ gates, Hadamard (H) gates, and CNOT gates.

According to the above discussion, it can be noticed that a COCNN analog to a QCNN is not easy to implement. So, we consider the simplification in the main text and perform the experiment.

**S4.** **The theoretical background of the experimental setup**

Our experimental aim is to realize the function of the quantum phase recognition circuit based on the QCNN. According to the QCNN scheme [3], the circuit of a 3-qubit QCNN for quantum phase recognition can be illustrated by Fig. S3. By using several algebraic tricks, the circuit can be transformed to a more practical one, as shown in Fig. S4.


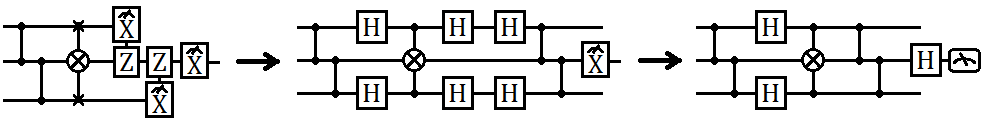


**Figure S3** 3-qubit QCNN for quantum phase recognition.

The simplification in Fig. S4 is done by using the following properties: 1) A Hadamard gate can rotate the qubit basis from $\left\{ \left| 0 \right\rangle,\left| 1 \right\rangle\right\}$ to $\left\{ \left| + \right\rangle,\left| - \right\rangle\right\}$. 2) The function of two Hadamard gates equals to the identity. 3) The measurement-based gate in the basis $\left\{ \left| + \right\rangle,\left| - \right\rangle\right\}$ equals to the circuit composed of a Hadamard gate, a CZ gate, and the action that leaves the control qubit away.


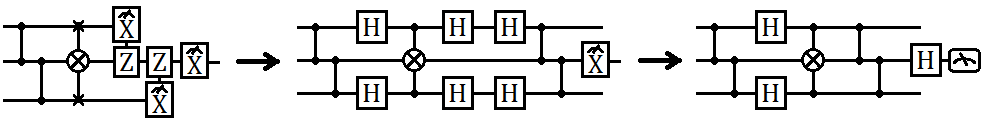
 **Figure S4** The simplification of procedure of circuit in Fig. S3.


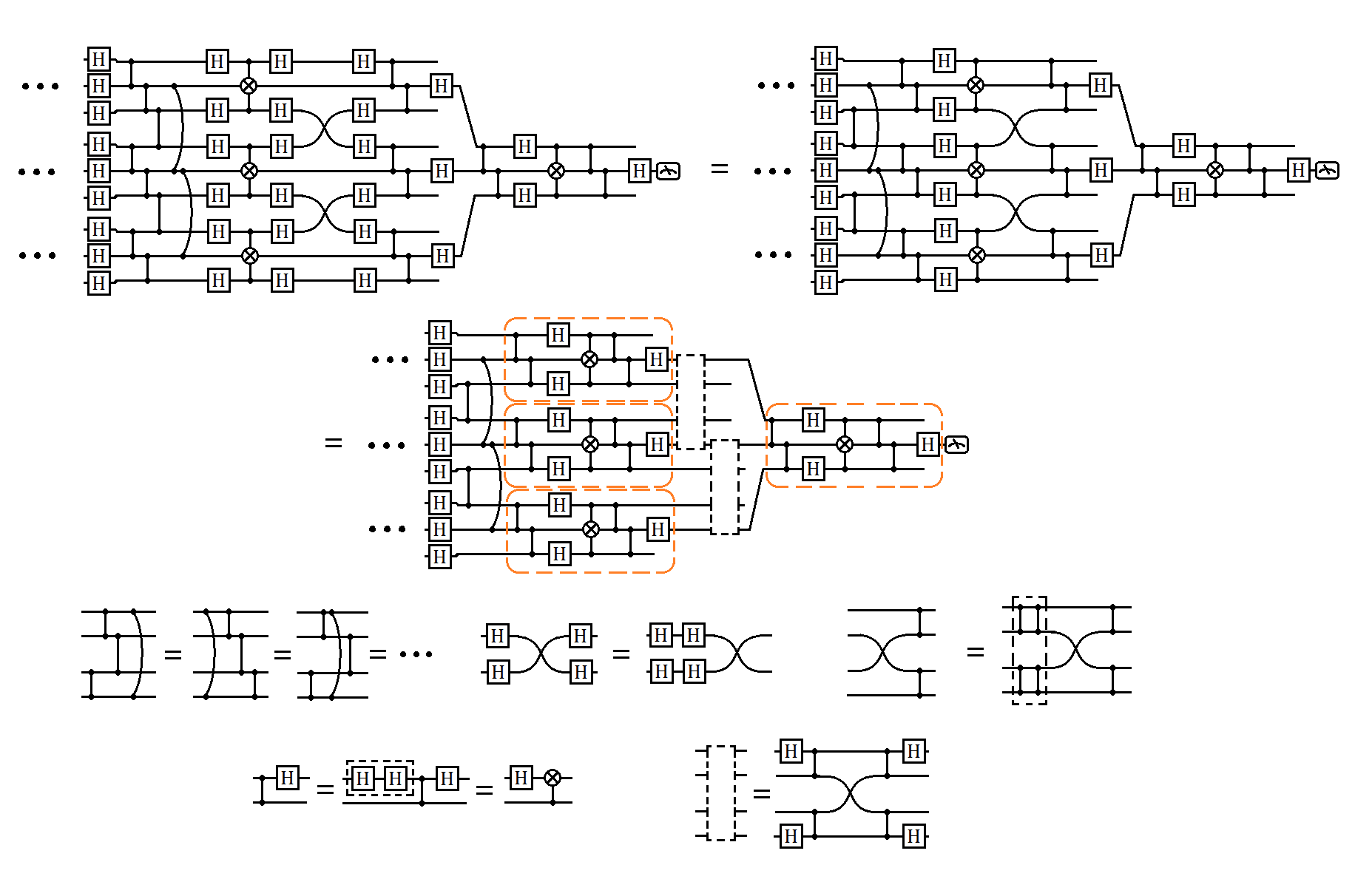


**Figure** **S5** The relation of the $N$-qubit quantum phase recognition circuit with the 3-qubit circuit (upper panel) and the tricks for obtaining the relation (lower panel).

In fact, the structure of the 3-qubit circuit in Fig. S3 is repetitively shown in the $N$-qubit quantum phase recognition circuit. This can be seen by the circuit relation in the upper panel of Fig. S5. The leftmost circuit is the original QCNN circuit phase recognition. By rearranging the gates, the original circuit can be transformed to the last circuit composed of the 3-qubit circuits (marked by the orange dashed square). The basic tricks for the transformation are shown below, and the instruction in the last is to explain the black dashed square in the last circuit. The most of the tricks are explained in the last paragraph, except the commutative and the orthonormal relation of the CZ gates. From the circuit relation, it indicates that the $N$-qubit quantum phase recognition circuit can be generated by the 3-qubit circuit in Fig. S3.

In our experiments, we consider to implement the 3-qubit circuit in Fig. S3. As we have discussed in the third section of the Materials and methods, the three beams $\boldsymbol{E}_{r}$, $\boldsymbol{E}_{s}$, and $\boldsymbol{E}_{t}$ in the scheme are set to be

$$1:\boldsymbol{E}_{r}=\boldsymbol{h}f_{1}+\boldsymbol{h}f_{2}+\boldsymbol{v}f_{3}+\boldsymbol{v}f_{4}$$

$$2:\boldsymbol{E}_{s}=\left( p_{s,1}^{H}\boldsymbol{h}+p_{s,1}^{V}\boldsymbol{v} \right)f_{1}+\left( p_{s,2}^{H}\boldsymbol{h}+p_{s,2}^{V}\boldsymbol{v} \right)f_{2}+\left( p_{s,3}^{H}\boldsymbol{h}+p_{s,3}^{V}\boldsymbol{v} \right)f_{3}+\left( p_{s,4}^{H}\boldsymbol{h}+p_{s,4}^{V}\boldsymbol{v} \right)f_{4}$$

$$3:\boldsymbol{E}_{t}=\boldsymbol{h}f_{1}+\boldsymbol{v}f_{2}+\boldsymbol{h}f_{3}+\boldsymbol{v}f_{4} \text{(}\text{S45}\text{)}$$

In M3, we have shown that the operations analog to the 3-qubit QCNN circuit can be implemented on the second beam $\boldsymbol{E}_{s}$. Also, the final output is given by the single beam $\boldsymbol{E}_{s}$, just like the single qubit output as shown in Fig. S3. So, we do not prepare the un-operated beam $\boldsymbol{E}_{r}$ and $\boldsymbol{E}_{t}$ in our experiment. We choose spatial modes to implement $f_{1}$ to $f_{4}$, and the four modes can be operated by Q-H-Qs for generating $\boldsymbol{E}_{s}$. Then, the operation analog to the quantum gates in Fig. S3 on $\boldsymbol{E}_{s}$ can be given step-by-step as follows.


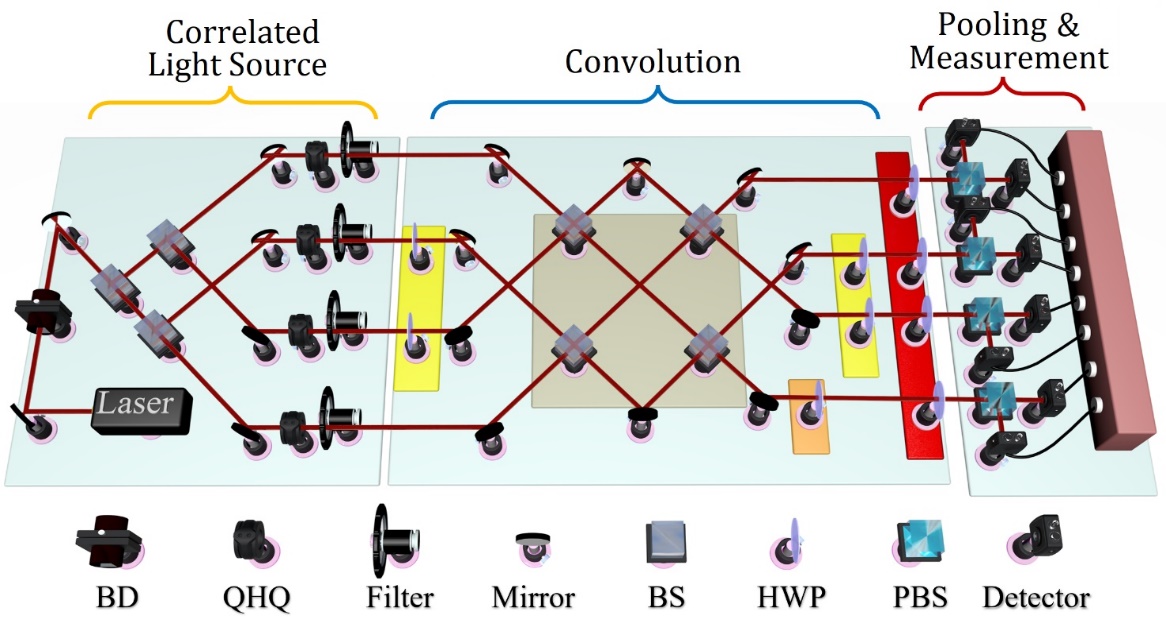


**Figure S6** Our experimental setup (the same with Fig. 3 in the main text).

1. The operation analog to the two CZ gates on the first pair of qubits and last pair of qubits.

In the qubit basis, the matrix of the two CZ gates $U_{CZ2}$ is expressed by

$$U_{\mathrm{CZ}2}=\left( U_{\mathrm{CZ}}\otimes I \right)\left( I\otimes U_{\mathrm{CZ}} \right)$$

$$=\left( \begin{matrix} 1 & 0 & 0 & 0 & 0 & 0 & 0 & 0 \\ 0 & 1 & 0 & 0 & 0 & 0 & 0 & 0 \\ 0 & 0 & 1 & 0 & 0 & 0 & 0 & 0 \\ 0 & 0 & 0 & -1 & 0 & 0 & 0 & 0 \\ 0 & 0 & 0 & 0 & 1 & 0 & 0 & 0 \\ 0 & 0 & 0 & 0 & 0 & 1 & 0 & 0 \\ 0 & 0 & 0 & 0 & 0 & 0 & -1 & 0 \\ 0 & 0 & 0 & 0 & 0 & 0 & 0 & 1 \end{matrix} \right)\text{ }\text{(}\text{S46}\text{)}$$

The matrix of the operation (in the basis $\left\{ \left| h_{1} \right)\left| h_{2} \right)\left| h_{3} \right),\left| h_{1} \right)\left| h_{2} \right)\left| v_{3} \right),\ldots,\left| v_{1} \right)\left| v_{2} \right)\left| v_{3} \right) \right\}$) analog to the two CZ gates is the same with Eq. (S46). Therefore, the matrix of this operation $M_{CZ2}$ in our experiments can be obtained by the strategy in M3,

$$M_{CZ2}=M_{s}^{-1}U_{\mathrm{CZ}2}M_{s}=\left( \begin{matrix} 1 & 0 & 0 & 0 & 0 & 0 & 0 & 0 \\ 0 & 1 & 0 & 0 & 0 & 0 & 0 & 0 \\ 0 & 0 & 1 & 0 & 0 & 0 & 0 & 0 \\ 0 & 0 & 0 & 1 & 0 & 0 & 0 & 0 \\ 0 & 0 & 0 & 0 & 1 & 0 & 0 & 0 \\ 0 & 0 & 0 & 0 & 0 & -1 & 0 & 0 \\ 0 & 0 & 0 & 0 & 0 & 0 & -1 & 0 \\ 0 & 0 & 0 & 0 & 0 & 0 & 0 & 1 \end{matrix} \right)\text{ }\text{(}\text{S47}\text{)}$$

Therefore, considering the order of the basis (as shown by $\left( p_{s,1}^{H} p_{s,2}^{H} p_{s,3}^{H} p_{s,4}^{H} p_{s,1}^{V} p_{s,2}^{V} p_{s,3}^{V} p_{s,4}^{V} \right)^{T}$), the form of $M_{\mathrm{CZ}2}$ means that this operation shifts the phase of the vertical polarization components of the mode $f_{2}$ and $f_{3}$ the by a factor of $\pi$. This can be implemented by the two HWPs oriented at the 0 rad, as shown by the left yellow region in Fig. S6.

1. The operation analog to two Hadamard gates on the first and the second qubit.

In the qubit basis, the matrix of the two Hadamard gates on the first and the second qubit, $U_{H2}$, is expressed by

$$U_{H2}=\frac{1}{2}\left( \begin{matrix} 1 & 1 & 0 & 0 & 1 & 1 & 0 & 0 \\ 1 & -1 & 0 & 0 & 1 & -1 & 0 & 0 \\ 0 & 0 & 1 & 1 & 0 & 0 & 1 & 1 \\ 0 & 0 & 1 & -1 & 0 & 0 & 1 & -1 \\ 1 & 1 & 0 & 0 & -1 & -1 & 0 & 0 \\ 1 & -1 & 0 & 0 & -1 & 1 & 0 & 0 \\ 0 & 0 & 1 & 1 & 0 & 0 & -1 & -1 \\ 0 & 0 & 1 & -1 & 0 & 0 & -1 & 1 \end{matrix} \right)\text{ }\text{(}\text{S48}\text{)}$$

Similar to (i), the matrix of the operation analog to the two Hadamard gates, $M_{H2}$, in our experiments can be obtained by

$$M_{H2}=M_{s}^{-1}U_{H2}M_{s}$$

$$=\frac{1}{2}\left( \begin{matrix} 1 & 1 & 1 & 1 & 0 & 0 & 0 & 0 \\ 1 & -1 & 1 & -1 & 0 & 0 & 0 & 0 \\ 1 & 1 & -1 & -1 & 0 & 0 & 0 & 0 \\ 1 & -1 & -1 & 1 & 0 & 0 & 0 & 0 \\ 0 & 0 & 0 & 0 & 1 & 1 & 1 & 1 \\ 0 & 0 & 0 & 0 & 1 & -1 & 1 & -1 \\ 0 & 0 & 0 & 0 & 1 & 1 & -1 & -1 \\ 0 & 0 & 0 & 0 & 1 & -1 & -1 & 1 \end{matrix} \right)\text{ }\text{(}\text{S49}\text{)}$$

The form of $M_{H2}$ means that this operation mixes the modes, which can be realized by the four beam splitters in the gray region in Fig S6. The instruction is given below.

As shown by Fig. S6, the left two BSs in the gray region mix the first and the third modes, and the second and the fourth modes respectively. The process can be characterized by

$$\left. \begin{matrix} f_{1}:p_{s,1}^{H}\boldsymbol{h}+p_{s,1}^{V}\boldsymbol{v} \\ f_{3}: p_{s,3}^{H}\boldsymbol{h}+p_{s,3}^{V}\boldsymbol{v} \end{matrix} \right\}\to\left\{ \begin{matrix} f_{1}:\frac{p_{s,1}^{H}+p_{s,3}^{H}}{\sqrt{2}}\boldsymbol{h}+\frac{p_{s,1}^{V}+p_{s,3}^{V}}{\sqrt{2}}\boldsymbol{v} \\ f_{3}: \frac{p_{s,1}^{H}-p_{s,3}^{H}}{\sqrt{2}}\boldsymbol{h}+\frac{p_{s,1}^{V}-p_{s,3}^{V}}{\sqrt{2}}\boldsymbol{v} \end{matrix} \right.$$

$$\left. \begin{matrix} f_{2}:p_{s,2}^{H}\boldsymbol{h}+p_{s,2}^{V}\boldsymbol{v} \\ f_{4}: p_{s,4}^{H}\boldsymbol{h}+p_{s,4}^{V}\boldsymbol{v} \end{matrix} \right\}\to\left\{ \begin{matrix} f_{2}:\frac{p_{s,2}^{H}+p_{s,4}^{H}}{\sqrt{2}}\boldsymbol{h}+\frac{p_{s,2}^{V}+p_{s,4}^{V}}{\sqrt{2}}\boldsymbol{v} \\ f_{4}: \frac{p_{s,2}^{H}-p_{s,4}^{H}}{\sqrt{2}}\boldsymbol{h}+\frac{p_{s,2}^{V}-p_{s,4}^{V}}{\sqrt{2}}\boldsymbol{v} \end{matrix} \right.\text{ }\text{(}\text{S50}\text{)}$$

Followingly, the right two BSs in the gray region mix the first and the third output of the left BSs, and the second and the fourth output of the left BSs, respectively.

$$\left. \begin{matrix} f_{1}:\frac{p_{s,1}^{H}+p_{s,3}^{H}}{\sqrt{2}}\boldsymbol{h}+\frac{p_{s,1}^{V}+p_{s,3}^{V}}{\sqrt{2}}\boldsymbol{v} \\ f_{2}: \frac{p_{s,2}^{H}+p_{s,4}^{H}}{\sqrt{2}}\boldsymbol{h}+\frac{p_{s,2}^{V}+p_{s,4}^{V}}{\sqrt{2}}\boldsymbol{v} \end{matrix} \right\}\to\left\{ \begin{matrix} f_{1}:\frac{p_{s,1}^{H}+p_{s,2}^{H}+p_{s,3}^{H}+p_{s,4}^{H}}{2}\boldsymbol{h}+\frac{p_{s,1}^{V}+p_{s,2}^{V}+p_{s,3}^{V}+p_{s,4}^{V}}{2}\boldsymbol{v} \\ f_{2}: \frac{p_{s,1}^{H}-p_{s,2}^{H}+p_{s,3}^{H}-p_{s,4}^{H}}{2}\boldsymbol{h}+\frac{p_{s,1}^{V}-p_{s,2}^{V}+p_{s,3}^{V}-p_{s,4}^{V}}{2}\boldsymbol{v} \end{matrix} \right.$$

$$\left. \begin{matrix} f_{3}:\frac{p_{s,1}^{H}-p_{s,3}^{H}}{\sqrt{2}}\boldsymbol{h}+\frac{p_{s,1}^{V}-p_{s,3}^{V}}{\sqrt{2}}\boldsymbol{v} \\ f_{4}: \frac{p_{s,2}^{H}-p_{s,4}^{H}}{\sqrt{2}}\boldsymbol{h}+\frac{p_{s,2}^{V}-p_{s,4}^{V}}{\sqrt{2}}\boldsymbol{v} \end{matrix} \right\}\to\left\{ \begin{matrix} f_{3}:\frac{p_{s,1}^{H}+p_{s,2}^{H}-p_{s,3}^{H}-p_{s,4}^{H}}{2}\boldsymbol{h}+\frac{p_{s,1}^{V}+p_{s,2}^{V}-p_{s,3}^{V}-p_{s,4}^{V}}{2}\boldsymbol{v} \\ f_{4}: \frac{p_{s,1}^{H}-p_{s,2}^{H}-p_{s,3}^{H}+p_{s,4}^{H}}{2}\boldsymbol{h}+\frac{p_{s,1}^{V}-p_{s,2}^{V}-p_{s,3}^{V}+p_{s,4}^{V}}{2}\boldsymbol{v} \end{matrix} \right.$$

$$\text{(}\text{S51}\text{)}$$

Therefore, by using a whole transformation matrix, Eq. ($\text{S50}$) and Eq. (S51) can be summarized by

$$\left( \begin{matrix} p_{s,1}^{H} \\ p_{s,2}^{H} \\ p_{s,3}^{H} \\ p_{s,4}^{H} \\ p_{s,1}^{V} \\ p_{s,2}^{V} \\ p_{s,3}^{V} \\ p_{s,4}^{V} \end{matrix} \right)\to\frac{1}{2}\left( \begin{matrix} p_{s,1}^{H}+p_{s,2}^{H}+p_{s,3}^{H}+p_{s,4}^{H} \\ p_{s,1}^{H}-p_{s,2}^{H}+p_{s,3}^{H}-p_{s,4}^{H} \\ p_{s,1}^{H}+p_{s,2}^{H}-p_{s,3}^{H}-p_{s,4}^{H} \\ p_{s,1}^{H}-p_{s,2}^{H}-p_{s,3}^{H}+p_{s,4}^{H} \\ p_{s,1}^{V}+p_{s,2}^{V}+p_{s,3}^{V}+p_{s,4}^{V} \\ p_{s,1}^{V}-p_{s,2}^{V}+p_{s,3}^{V}-p_{s,4}^{V} \\ p_{s,1}^{V}+p_{s,2}^{V}-p_{s,3}^{V}-p_{s,4}^{V} \\ p_{s,1}^{V}-p_{s,2}^{V}-p_{s,3}^{V}+p_{s,4}^{V} \end{matrix} \right)=\frac{1}{2}\left( \begin{matrix} 1 & 1 & 1 & 1 & 0 & 0 & 0 & 0 \\ 1 & -1 & 1 & -1 & 0 & 0 & 0 & 0 \\ 1 & 1 & -1 & -1 & 0 & 0 & 0 & 0 \\ 1 & -1 & -1 & 1 & 0 & 0 & 0 & 0 \\ 0 & 0 & 0 & 0 & 1 & 1 & 1 & 1 \\ 0 & 0 & 0 & 0 & 1 & -1 & 1 & -1 \\ 0 & 0 & 0 & 0 & 1 & 1 & -1 & -1 \\ 0 & 0 & 0 & 0 & 1 & -1 & -1 & 1 \end{matrix} \right)\left( \begin{matrix} p_{s,1}^{H} \\ p_{s,2}^{H} \\ p_{s,3}^{H} \\ p_{s,4}^{H} \\ p_{s,1}^{V} \\ p_{s,2}^{V} \\ p_{s,3}^{V} \\ p_{s,4}^{V} \end{matrix} \right)\text{ }\text{(}\text{S52}\text{)}$$

which is exactly required by Eq. (S49).

1. The operation analog to the Toffoli gate with the second qubit being the target qubit.

In the qubit basis, the matrix of the Toffoli gate with the second qubit being the target qubit, $U_{\mathrm{Toffoli}}$, is expressed by

$$U_{\mathrm{Toffoli}}=\left( \begin{matrix} 1 & 0 & 0 & 0 & 0 & 0 & 0 & 0 \\ 0 & 1 & 0 & 0 & 0 & 0 & 0 & 0 \\ 0 & 0 & 1 & 0 & 0 & 0 & 0 & 0 \\ 0 & 0 & 0 & 1 & 0 & 0 & 0 & 0 \\ 0 & 0 & 0 & 0 & 1 & 0 & 0 & 0 \\ 0 & 0 & 0 & 0 & 0 & 0 & 0 & 1 \\ 0 & 0 & 0 & 0 & 0 & 0 & 1 & 0 \\ 0 & 0 & 0 & 0 & 0 & 1 & 0 & 0 \end{matrix} \right) \text{(}\text{S53}\text{)}$$

Using the above strategy, the matrix of the operation analog to the two Hadamard gates, $M_{\mathrm{Toffoli}}$, in our experiments can be given by

$$M_{\mathrm{Toffoli}}=M_{s}^{-1}U_{\mathrm{Toffoli}}M_{s}$$

$$=\left( \begin{matrix} 1 & 0 & 0 & 0 & 0 & 0 & 0 & 0 \\ 0 & 1 & 0 & 0 & 0 & 0 & 0 & 0 \\ 0 & 0 & 1 & 0 & 0 & 0 & 0 & 0 \\ 0 & 0 & 0 & 0 & 0 & 0 & 0 & 1 \\ 0 & 0 & 0 & 0 & 1 & 0 & 0 & 0 \\ 0 & 0 & 0 & 0 & 0 & 1 & 0 & 0 \\ 0 & 0 & 0 & 0 & 0 & 0 & 1 & 0 \\ 0 & 0 & 0 & 1 & 0 & 0 & 0 & 0 \end{matrix} \right)\text{ }\text{(}\text{S54}\text{)}$$

The form of $M_{\mathrm{Toffoli}}$ means that this operation exchanges the horizontal and vertical components of the mode $f_{4}$. This can be implemented by an HWP oriented at $\pi/4$, as shown by the orange region in Fig. S6.

1. The operation analog to the two CZ gates on the first pair of qubits and last pair of qubits.

The operation in this step is the same with that given by (i). It is also implemented by the two HWPs oriented at the 0 rad, as shown by the right yellow region in Fig. S6.

1. The operation analog to a single Hadamard gate on the second qubit.

In the qubit basis, the matrix of the single Hadamard gate on the second qubit, $U_{H1}$, is expressed by

$$U_{H1}=\frac{1}{\sqrt{2}}\left( \begin{matrix} 1 & 0 & 1 & 0 & 0 & 0 & 0 & 0 \\ 0 & 1 & 0 & 1 & 0 & 0 & 0 & 0 \\ 1 & 0 & -1 & 0 & 0 & 0 & 0 & 0 \\ 0 & 1 & 0 & -1 & 0 & 0 & 0 & 0 \\ 0 & 0 & 0 & 0 & 1 & 0 & 1 & 0 \\ 0 & 0 & 0 & 0 & 0 & 1 & 0 & 1 \\ 0 & 0 & 0 & 0 & 1 & 0 & -1 & 0 \\ 0 & 0 & 0 & 0 & 0 & 1 & 0 & -1 \end{matrix} \right) \text{(}\text{S55}\text{)}$$

Using the above strategy, the matrix of the operation analog to the single Hadamard gate, $M_{H1}$, in our experiments can be given by

$$M_{H1}=M_{s}^{-1}U_{H1}M_{s}$$

$$=\frac{1}{\sqrt{2}}\left( \begin{matrix} 1 & 0 & 0 & 0 & 1 & 0 & 0 & 0 \\ 0 & 1 & 0 & 0 & 0 & 1 & 0 & 0 \\ 0 & 0 & 1 & 0 & 0 & 0 & 1 & 0 \\ 0 & 0 & 0 & 1 & 0 & 0 & 0 & 1 \\ 1 & 0 & 0 & 0 & -1 & 0 & 0 & 0 \\ 0 & 1 & 0 & 0 & 0 & -1 & 0 & 0 \\ 0 & 0 & 1 & 0 & 0 & 0 & -1 & 0 \\ 0 & 0 & 0 & 1 & 0 & 0 & 0 & -1 \end{matrix} \right) \text{(}\text{S56}\text{)}$$

The form of $M_{H1}$ means that this operation transforms the horizontal components of the four modes (from $f_{1}$ to $f_{4}$) to the equal-weighted sum of their horizontal and vertical components respectively, and transforms the vertical components of the four modes to the qual-weighted difference of their horizontal and vertical components respectively. This can be implemented by four HWPs oriented at $\pi/8$ on each mode, as shown by the red region in Fig. S6.

By the steps (i)-(v), we show that the operations in Fig. S6 (or Fig. 3 in the main text) can perform the function of the circuit shown by Fig. S3. Next, we discuss the measurements of the output. The original quantum circuit given by Fig. S3 indicates that the output of the circuit is the single qubit state obtained by leaving the first and the third qubit of the 3-qubit state away. If we denote the 3-qubit state by $\left| \psi_{3} \right\rangle$ (Eq. (15) in the main text, which is also applied in section S2), the output can be given by

$$\rho_{2}=\mathrm{Tr}_{1,3}\left\{ \left| \psi_{3} \right\rangle\left\langle\psi_{3} \right| \right\}=\left( \left\langle0 \right|\otimes I\otimes\left\langle0 \right| \right)\left| \psi_{3} \right\rangle\left\langle\psi_{3} \right|\left( \left| 0 \right\rangle\otimes I\otimes\left| 0 \right\rangle\right)+\left( \left\langle0 \right|\otimes I\otimes\left\langle1 \right| \right)\left| \psi_{3} \right\rangle\left\langle\psi_{3} \right|\left( \left| 0 \right\rangle\otimes I\otimes\left| 1 \right\rangle\right)+\left( \left\langle1 \right|\otimes I\otimes\left\langle0 \right| \right)\left| \psi_{3} \right\rangle\left\langle\psi_{3} \right|\left( \left| 1 \right\rangle\otimes I\otimes\left| 0 \right\rangle\right)+\left( \left\langle1 \right|\otimes I\otimes\left\langle1 \right| \right)\left| \psi_{3} \right\rangle\left\langle\psi_{3} \right|\left( \left| 1 \right\rangle\otimes I\otimes\left| 1 \right\rangle\right)=\left( q_{000}q_{000}^{*}+q_{001}q_{001}^{*}+q_{100}q_{100}^{*}+q_{101}q_{101}^{*} \right)\left| 0 \right\rangle\left\langle0 \right|+\left( q_{000}q_{010}^{*}+q_{001}q_{011}^{*}+q_{100}q_{110}^{*}+q_{101}q_{111}^{*} \right)\left| 0 \right\rangle\left\langle1 \right|+\left( q_{010}q_{000}^{*}+q_{011}q_{001}^{*}+q_{110}q_{100}^{*}+q_{111}q_{101}^{*} \right)\left| 1 \right\rangle\left\langle0 \right|+\left( q_{010}q_{010}^{*}+q_{011}q_{011}^{*}+q_{110}q_{110}^{*}+q_{111}q_{111}^{*} \right)\left| 1 \right\rangle\left\langle1 \right|$$

$$\text{(}\text{S5}\text{7}\text{)}$$

One way to obtain the complete information of the output state is the Pauli measurements, given by

$$\mathrm{Tr}\left\{ X\rho_{2} \right\}=2\mathrm{Re}\left\{ q_{000}q_{010}^{*}+q_{001}q_{011}^{*}+q_{100}q_{110}^{*}+q_{101}q_{111}^{*} \right\},$$

$$\mathrm{Tr}\left\{ Y\rho_{2} \right\}=2\mathrm{Im}\left\{ q_{000}q_{010}^{*}+q_{001}q_{011}^{*}+q_{100}q_{110}^{*}+q_{101}q_{111}^{*} \right\},$$

$$\mathrm{Tr}\left\{ Z\rho_{2} \right\}=\left( \left| q_{000} \right|^{2}+\left| q_{001} \right|^{2}+\left| q_{100} \right|^{2}+\left| q_{101} \right|^{2} \right)-\left( \left| q_{010} \right|^{2}+\left| q_{011} \right|^{2}+\left| q_{110} \right|^{2}+\left| q_{111} \right|^{2} \right)$$

$$\text{(}\text{S5}\text{8}\text{)}$$

In our setup, according to Eq. (19)-(20) in the main text, one has $M_{s}\left( p_{s,1}^{H} p_{s,2}^{H} p_{s,3}^{H} p_{s,4}^{H} p_{s,1}^{V} p_{s,2}^{V} p_{s,3}^{V} p_{s,4}^{V} \right)^{T}=\left( q_{000} q_{001} q_{010} q_{011} q_{100} q_{101} q_{110} q_{111} \right)^{T}$. Therefore, the following relation can be obtained,

$$p_{s,1}^{H}=q_{000},p_{s,2}^{H}=q_{001},p_{s,3}^{H}=q_{100},p_{s,4}^{H}=q_{101}$$

$$p_{s,1}^{V}=q_{010},p_{s,2}^{V}=q_{011},p_{s,3}^{V}=q_{110},p_{s,4}^{V}=q_{111} \text{(}\text{S5}\text{9}\text{)}$$

The measurements analog to the Pauli measurement can be given below. First, if one applies an HWP oriented at $\pi/8$to rotate the polarization of each of the four modes, it can be obtained that

$$\left( \begin{matrix} p_{s,1}^{H} \\ p_{s,2}^{H} \\ p_{s,3}^{H} \\ p_{s,4}^{H} \\ p_{s,1}^{V} \\ p_{s,2}^{V} \\ p_{s,3}^{V} \\ p_{s,4}^{V} \end{matrix} \right)\to\frac{1}{\sqrt{2}}\left( \begin{matrix} p_{s,1}^{H}+p_{s,1}^{V} \\ p_{s,2}^{H}+p_{s,2}^{V} \\ p_{s,3}^{H}+p_{s,3}^{V} \\ p_{s,4}^{H}+p_{s,4}^{V} \\ p_{s,1}^{H}-p_{s,1}^{V} \\ p_{s,2}^{H}-p_{s,2}^{V} \\ p_{s,3}^{H}-p_{s,3}^{V} \\ p_{s,4}^{H}-p_{s,4}^{V} \end{matrix} \right)\text{ }\text{(}\text{S60}\text{)}$$

Measuring the difference of the horizontal and vertical components after the rotation, one has

$$M_{X}=\left| p_{s,1}^{H}\boldsymbol{+}p_{s,1}^{V} \right|^{2}+\left| p_{s,2}^{H}\boldsymbol{+}p_{s,2}^{V} \right|^{2}+\left| p_{s,3}^{H}\boldsymbol{+}p_{s,3}^{V} \right|^{2}+\left| p_{s,4}^{H}\boldsymbol{+}p_{2,4}^{V} \right|^{2}-\left| p_{2,1}^{H}\boldsymbol{-}p_{2,1}^{V} \right|^{2}-\left| p_{2,2}^{H}\boldsymbol{-}p_{2,2}^{V} \right|^{2}-\left| p_{2,3}^{H}\boldsymbol{-}p_{2,3}^{V} \right|^{2}+\left| p_{2,4}^{H}\boldsymbol{-}p_{2,4}^{V} \right|^{2}$$

$$\propto\mathrm{Re}\left\{ q_{000}q_{010}^{*}+q_{001}q_{011}^{*}+q_{100}q_{110}^{*}+q_{101}q_{111}^{*} \right\}=\mathrm{Tr}\left\{ X\rho_{2} \right\} \text{(}\text{S6}\text{1}\text{)}$$

which corresponds to the Pauli-*X* measurement of the qubit. Second, if one applies a QWP oriented at $\pi/8$to each of the four modes, it can be obtained that

$$\left( \begin{matrix} p_{s,1}^{H} \\ p_{s,2}^{H} \\ p_{s,3}^{H} \\ p_{s,4}^{H} \\ p_{s,1}^{V} \\ p_{s,2}^{V} \\ p_{s,3}^{V} \\ p_{s,4}^{V} \end{matrix} \right)\to\frac{1}{\sqrt{2}}\left( \begin{matrix} p_{s,1}^{H}+ip_{s,1}^{V} \\ p_{s,2}^{H}+ip_{s,2}^{V} \\ p_{s,3}^{H}+ip_{s,3}^{V} \\ p_{s,4}^{H}+ip_{s,4}^{V} \\ p_{s,1}^{H}-ip_{s,1}^{V} \\ p_{s,2}^{H}-ip_{s,2}^{V} \\ p_{s,3}^{H}-ip_{s,3}^{V} \\ p_{s,4}^{H}-ip_{s,4}^{V} \end{matrix} \right) \text{(}\text{S6}\text{2}\text{)}$$

Measuring the difference of the horizontal and vertical components thereafter, one has

$$M_{Y}=\left| p_{2,1}^{H}\boldsymbol{+}{ip}_{2,1}^{V} \right|^{2}+\left| p_{2,2}^{H}\boldsymbol{+}ip_{2,2}^{V} \right|^{2}+\left| p_{2,3}^{H}\boldsymbol{+}ip_{2,3}^{V} \right|^{2}+\left| p_{2,4}^{H}\boldsymbol{+}ip_{2,4}^{V} \right|^{2}-\left| p_{2,1}^{H}\boldsymbol{-}ip_{2,1}^{V} \right|^{2}+\left| p_{2,2}^{H}\boldsymbol{-}ip_{2,2}^{V} \right|^{2}+\left| p_{2,3}^{H}\boldsymbol{-}ip_{2,3}^{V} \right|^{2}+\left| p_{2,4}^{H}\boldsymbol{-}ip_{2,4}^{V} \right|^{2}$$

$$\propto\mathrm{Im}\left\{ q_{000}q_{010}^{*}+q_{001}q_{011}^{*}+q_{100}q_{110}^{*}+q_{101}q_{111}^{*} \right\}=\mathrm{Tr}\left\{ Y\rho_{2} \right\} \text{(}\text{S6}\text{3}\text{)}$$

which corresponds to the Pauli-*Y* measurement of the qubit. Third, if one does nothing to the modes, and measure the difference of the horizontal and vertical components, it can be obtained that

$$M_{Y}=\left| p_{2,1}^{H} \right|^{2}+\left| p_{2,2}^{H} \right|^{2}+\left| p_{2,3}^{H} \right|^{2}+\left| p_{2,4}^{H} \right|^{2}-\left| p_{2,1}^{V} \right|^{2}-\left| p_{2,2}^{V} \right|^{2}-\left| p_{2,3}^{V} \right|^{2}-\left| p_{2,4}^{V} \right|^{2}$$

$$\propto\left( \left| q_{000} \right|^{2}+\left| q_{001} \right|^{2}+\left| q_{100} \right|^{2}+\left| q_{101} \right|^{2} \right)-\left( \left| q_{010} \right|^{2}+\left| q_{011} \right|^{2}+\left| q_{110} \right|^{2}+\left| q_{111} \right|^{2} \right)=\mathrm{Tr}\left\{ Z\rho_{2} \right\}\text{ }\text{ }\text{(}\text{S6}\text{4}\text{)}$$

which corresponds to the Pauli-*Z* measurement of the qubit. In summary, the measurement analog to the Pauli measurements can be performed for the beam state. $M_{X}$, $M_{Y}$, and $M_{Z}$ also equal to the term $\mathrm{Tr}\left\{ X\left| E_{out} \right)\left( E_{out} \right| \right\}$, $\mathrm{Tr}\left\{ Y\left| E_{out} \right)\left( E_{out} \right| \right\}$, and $\mathrm{Tr}\left\{ Z\left| E_{out} \right)\left( E_{out} \right| \right\}$ respectively. Besides, it is worth noticing that the quantum results of Eq. (S57) are the ones obtained after the pooling. The measurement relations Eq. (S61), (S63), and (S64) shows that they can provide the information analog to the quantum results after the pooling. Hence, the part of pooling in the experiments is indirectly performed by the final measurements. The experimental results in the main text are measured by using the above relation Eq. (S61), (S63), and (S64).

**S5. The theoretical calculation of the experimental output for the ten input states considered in the main text**

As mentioned in the main text, we experimentally check the output of our setup shown by Fig. S6 by setting the input to be 10 states, each of which is analog to a 3-qubit quantum states. Given an arbitrary 3-qubit state, or the particularly amplitude of the basis $\left( q_{000} q_{001} q_{010} q_{011} q_{100} q_{101} q_{110} q_{111} \right)^{T}$, the parameters of the mode $f_{1}$ to $f_{4}$ can be obtained by

$$\left( \begin{matrix} p_{s,1}^{H} \\ p_{s,2}^{H} \\ p_{s,3}^{H} \\ p_{s,4}^{H} \\ p_{s,1}^{V} \\ p_{s,2}^{V} \\ p_{s,3}^{V} \\ p_{s,4}^{V} \end{matrix} \right)=\left( \begin{matrix} 1 & 0 & 0 & 0 & 0 & 0 & 0 & 0 \\ 0 & 1 & 0 & 0 & 0 & 0 & 0 & 0 \\ 0 & 0 & 0 & 0 & 1 & 0 & 0 & 0 \\ 0 & 0 & 0 & 0 & 0 & 1 & 0 & 0 \\ 0 & 0 & 1 & 0 & 0 & 0 & 0 & 0 \\ 0 & 0 & 0 & 1 & 0 & 0 & 0 & 0 \\ 0 & 0 & 0 & 0 & 0 & 0 & 1 & 0 \\ 0 & 0 & 0 & 0 & 0 & 0 & 0 & 1 \end{matrix} \right)\left( \begin{matrix} q_{000} \\ q_{001} \\ q_{010} \\ q_{011} \\ q_{100} \\ q_{101} \\ q_{110} \\ q_{111} \end{matrix} \right)\text{ }\text{(}\text{S65}\text{)}$$

By using the measurement relation Eq. (S61), (S63), and (S64), the considered quantum states, the corresponding beam setups, the theoretical measurement results of the beam states, and the corresponding density matrix form of the output beam state are given by Table S2. As discussed in the main text, the density matrix form here is an analogy of the quantum density matrix, obtained by $M_{I}I+M_{X}X+M_{X}X+M_{Z}Z$, where $M_{I}$ is the intensity sum of all the components. $M_{I}$ corresponds to $\mathrm{Tr}\left\{ \left| E_{out} \right)\left( E_{out} \right| \right\}$ or $\mathrm{Tr}\left\{ \rho_{2} \right\}$. For the experimental data, $M_{I}$ is also used for normalization.

In Table S2, $\left| L \right\rangle=\left( \left| 0 \right\rangle-i\left| 1 \right\rangle\right)/\sqrt{2}$，$\left| R \right\rangle=\left( \left| 0 \right\rangle+i\left| 1 \right\rangle\right)/\sqrt{2}$，$\left| \mathrm{GHZ} \right\rangle=\left( \left| 000 \right\rangle+\left| 111 \right\rangle\right)/\sqrt{2}$ and $\left| W \right\rangle=\left( \left| 001 \right\rangle+\left| 010 \right\rangle+\left| 100 \right\rangle\right)/\sqrt{3}$. The experimental results of these examples are shown by the Fig. 4 in the main text. It can be observed that the experimental results match well with the theoretical results given by the last column of Table S2.

**Table S2** The beam setup for mimicking the quantum states and the theoretical measurements of the outputs.

| Quantum states | Corresponding beam setup | Three measurements of the output | Density matrix form of the output |
| --- | --- | --- | --- |
| $\left\vert000 \right\rangle$ | $f_{1}\boldsymbol{h}$ | $M_{X}=0,M_{Y}=0,$  $M_{Z}=0.5$ | $\left( \begin{matrix} 0.75 & 0 \\ 0 & 0.25 \end{matrix} \right)$ |
| $\left\vert100 \right\rangle$ | $f_{3}\boldsymbol{h}$ | $M_{X}=0,M_{Y}=0,$  $M_{Z}=0.5$ | $\left( \begin{matrix} 0.75 & 0 \\ 0 & 0.25 \end{matrix} \right)$ |
| $\left\vert110 \right\rangle$ | $f_{3}\boldsymbol{v}$ | $M_{X}=0,M_{Y}=0,$  $M_{Z}=-0.5$ | $\left( \begin{matrix} 0.25 & 0 \\ 0 & 0.75 \end{matrix} \right)$ |
| $\left\vert111 \right\rangle$ | $f_{4}\boldsymbol{v}$ | $M_{X}=0,M_{Y}=0,$  $M_{Z}=-0.5$ | $\left( \begin{matrix} 0.25 & 0 \\ 0 & 0.75 \end{matrix} \right)$ |
| $\left\vert+-+ \right\rangle$ | $\frac{\sqrt{2}}{4}\left( f_{1}+f_{2}+f_{3}+f_{4} \right)\left( \boldsymbol{h}-\boldsymbol{v} \right)$ | $M_{X}=0,M_{Y}=0,$  $M_{Z}=1$ | $\left( \begin{matrix} 1 & 0 \\ 0 & 0 \end{matrix} \right)$ |
| $\left\vert-+- \right\rangle$ | $\frac{\sqrt{2}}{4}\left( f_{1}-f_{2}-f_{3}+f_{4} \right)\left( \boldsymbol{h}+\boldsymbol{v} \right)$ | $M_{X}=0,M_{Y}=0,$  $M_{Z}=-1$ | $\left( \begin{matrix} 0 & 0 \\ 0 & 1 \end{matrix} \right)$ |
| $\left\vert LRL \right\rangle$ | $\frac{\sqrt{2}}{4}\left( f_{1}\boldsymbol{-}if_{2}-if_{3}-f_{4} \right)\left( \boldsymbol{h}\boldsymbol{+}\boldsymbol{iv} \right)$ | $M_{X}=0,M_{Y}=0.5,$  $M_{Z}=0$ | $\left( \begin{matrix} 0.5 & 0.25i \\ -0.25i & 0.5 \end{matrix} \right)$ |
| $\left\vert RLR \right\rangle$ | $\frac{\sqrt{2}}{4}\left( f_{1}+if_{2}+if_{3}-f_{4} \right)\left( \boldsymbol{h}\boldsymbol{-}\boldsymbol{iv} \right)$ | $M_{X}=0,M_{Y}=-0.5,$  $M_{Z}=0$ | $\left( \begin{matrix} 0.5 & -0.25i \\ 0.25i & 0.5 \end{matrix} \right)$ |
| $\left\vert GHZ \right\rangle$ | $\frac{\sqrt{2}}{2}\left( f_{1}\boldsymbol{h}\boldsymbol{+}f_{4}\boldsymbol{v} \right)$ | $M_{X}=1,M_{Y}=0,M_{Z}=0$ | $\left( \begin{matrix} 0.5 & 0.5 \\ 0.5 & 0.5 \end{matrix} \right)$ |
| $\left\vert W \right\rangle$ | $\frac{\sqrt{3}}{3}\left( f_{1}\boldsymbol{v}\boldsymbol{+}f_{2}\boldsymbol{h}\boldsymbol{+}f_{3}\boldsymbol{h} \right)$ | $M_{X}=0,M_{Y}=0,$  $M_{Z}=-0.1667$ | $\left( \begin{matrix} 0.4167 & 0 \\ 0 & 0.5833 \end{matrix} \right)$ |

**S6. The data for giving the results of the phase recognition**

The main function of the circuit illustrated by Fig. S3 is to effectively judging the quantum phase of the input 3-qubit state by the output single qubit state. In order to show the recognition by our setup, we encode the ground state of the 3-site Haldane Hamiltonian and then measure the $M_{Z}$ of output. Particularly, the 3-site Haldane Hamiltonian can be given by

$$H=-JZ_{1}X_{2}Z_{3}-h_{1}\left( X_{1}+X_{2}+X_{3} \right)-h_{2}\left( X_{1}X_{2}+X_{2}X_{3} \right)\text{ }\text{(}\text{S66}\text{)}$$

The ground states of the Hamiltonian under a set of the parameters $J$, $h_{1}$, and $h_{2}$ can be calculated by the diagonalization. After the diagonalization, the amplitude of the basis $\left( q_{000} q_{001} q_{010} q_{011} q_{100} q_{101} q_{110} q_{111} \right)^{T}$ of the ground states can be obtained. Then, the beam setup can be given by using Eq. (S65). The experimental results after the normalization are provided by Table S3.

**Table S3** The experimental results of $M_{Z}$ when the inputs are the beam states encoding the ground states of the Haldane Hamiltonian under certain parameters. Each result is measured for five times and the variance is marked by the value in the bracket.

| $h_{1}/J$ | $h_{2}/J$ | 0.4 | 0.8 | 1.2 | 1.6 |
| --- | --- | --- | --- | --- | --- |
| -2.0 | | 0.1809 (0.0090) | 0.1948(0.0120) | 0.1247(0.0125) | 0.1194(0.0038) |
| -1.75 | | 0.1956 (0.0024) | 0.2096(0.0034) | 0.1388(0.0039) | 0.1254(0.0094) |
| -1.5 | | 0.2511(0.0046) | 0.2092(0.0069) | 0.1918(0.0095) | 0.1930(0.0064) |
| -1.25 | | 0.2617 (0.0067) | 0.2328(0.0069) | 0.1767(0.0056) | 0.1822(0.0028) |
| -1.0 | | 0.3399 (0.0039) | 0.2874(0.0086) | 0.2156(0.0031) | 0.1940(0.0063) |
| -0.75 | | 0.4094 (0.0020) | 0.3290(0.0016) | 0.2050(0.0085) | 0.5121(0.0067) |
| -0.5 | | 0.4735 (0.0042) | 0.3929(0.0090) | 0.5325(0.0014) | 0.4233(0.0099) |
| -0.25 | | 0.9016 (0.0065) | 0.7038 (0.0080) | 0.4045(0.0054) | 0.3339(0.0097) |
| 0 | | 0.7414 (0.0056) | 0.5795(0.0111) | 0.3777(0.0117) | 0.2924(0.0079) |
| 0.25 | | 0.6401 (0.0054) | 0.4107(0.0073) | 0.3281(0.0062) | 0.2590(0.0039) |
| 0.5 | | 0.4421 (0.0060) | 0.3607 (0.0075) | 0.2608(0.0032) | 0.2295(0.0031) |
| 0.75 | | 0.4427 (0.0083) | 0.3047(0.0082) | 0.2158(0.0041) | 0.1892(0.0057) |
| 1 | | 0.3436(0.0051) | 0.2904(0.0034) | 0.1959(0.0058) | 0.1840(0.0099) |
| 1.25 | | 0.3225(0.0022) | 0.2972(0.0101) | 0.1747(0.0029) | 0.1687(0.0026) |
| 1.5 | | 0.2750(0.0062) | 0.2560(0.0076) | 0.1516(0.0026) | 0.1685(0.0131) |
| 1.75 | | 0.2209 (0.0056) | 0.2350(0.0102) | 0.1461(0.0027) | 0.1599(0.0044) |
| 2.0 | | 0.2323(0.0046) | 0.2290(0.0066) | 0.1922(0.0038) | 0.1360(0.0081) |

The left panel of Fig. 5 is plotted by the data of Table S3. From the left panel of Fig. 5, we show that the experimental data matches well with the curves obtained by the standard method (string-order parameters). The boundaries of the phases are obtained by the second order derivative of the experimental data, as those instructed in the main text.

**References**

[1] M. A. Nielsen and I. L. Chuang, *Quantum Computation and Quantum Information*, 10th anniversary ed (Cambridge University Press, Cambridge ; New York, 2010).

[2] Y. Sun, Q. Li, L. Kong, J. Shang, and X. Zhang, *Universal Classical Optical Computing Inspired by Quantum Information Process*, Annalen Der Physik **534**, 2200360 (2022).

[3] I. Cong, S. Choi, and M. D. Lukin, *Quantum Convolutional Neural Networks*, Nat. Phys. **15**, 1273 (2019).

[4] B. N. Simon, C. M. Chandrashekar, and S. Simon, *Hamilton’s Turns as a Visual Tool Kit for Designing Single-Qubit Unitary Gates*, Phys. Rev. A **85**, 022323 (2012).
